# Emergent Pneumonia in Children

**DOI:** 10.3389/fped.2021.676296

**Published:** 2021-06-17

**Authors:** Cecilia Perret, Nicole Le Corre, Jose A. Castro-Rodriguez

**Affiliations:** ^1^Department of Pediatric Infectious Diseases and Immunology, School of Medicine, Pontificia Universidad Católica de Chile, Santiago, Chile; ^2^Department of Pediatric Pulmonology and Cardiology, School of Medicine, Pontificia Universidad Católica de Chile, Santiago, Chile

**Keywords:** pneumonia - clinical features and management, children, emerging respiratory pathogens, re-emerging respiratory pathogens, COVID - 19

## Abstract

In recent decades there have been multiple pathogens, viruses and bacteria, which have emerged as causal agents of pneumonia affecting adults, albeit less frequently, to children. For the purposes of this article we have classified emerging pathogens as follows: **True emerging**, to pathogens identified for the very first time affecting human population (SARS-CoV-1, SARS-CoV-2, MERS-CoV, avian influenza, and hantavirus); **Re-emerging**, to known pathogens which circulation was controlled once, but they have reappeared (measles, tuberculosis, antimicrobial resistant bacteria such as *CA-MRSA, Mycoplasma pneumoniae, Acinetobacter baumannii, Pseudomonas aeruginosa, Stenotrophomonas maltophilia*, and new serotypes of post-vaccine pneumococcal); and finally, those that we have called **old known with new presentations**, including common pathogens that, in particular condition, have changed their form of presentation (rhinovirus, and non-SARS coronavirus). We will review for each of them their epidemiology, forms of presentation, therapy, and prognosis in children compared to the adult with the aim of being able to recognize them to establish appropriate therapy, prognostics, and effective control measures.

## Introduction

**Figure 1:**
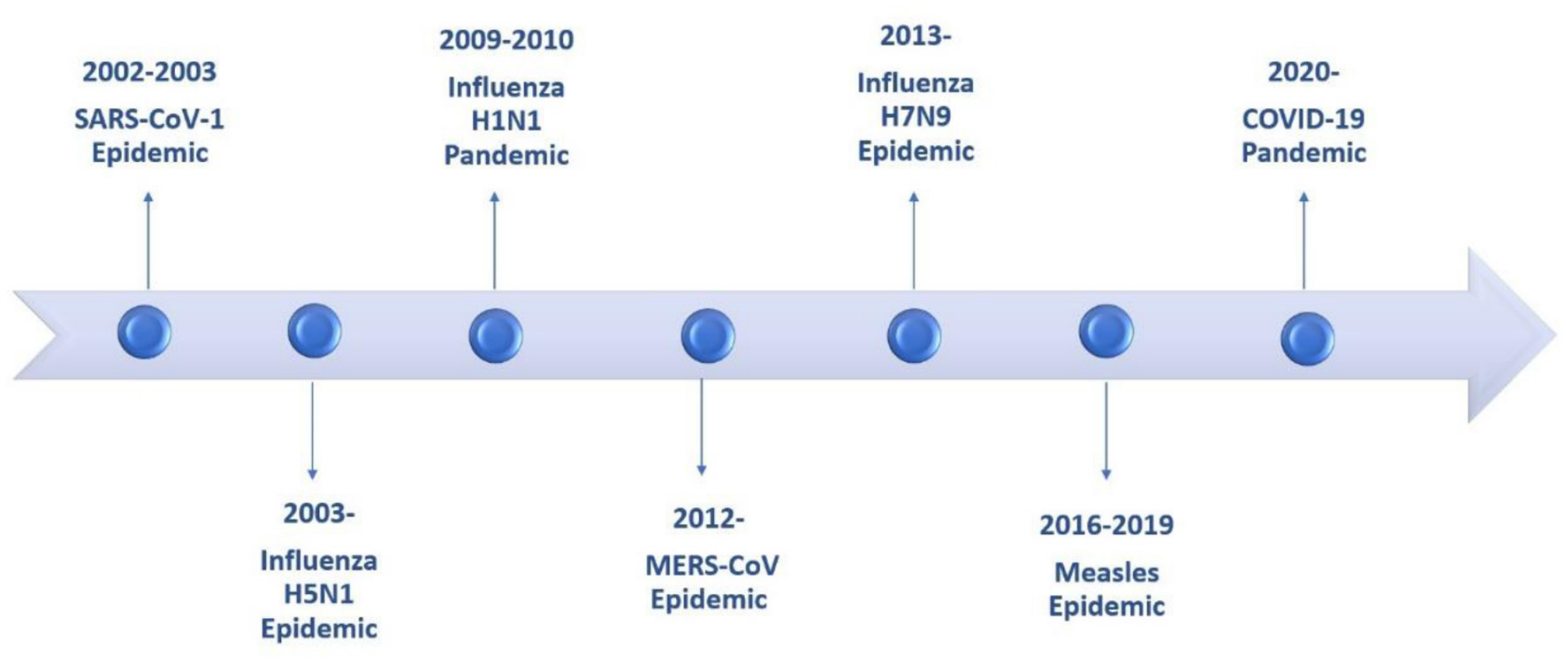
Timeline of epidemics and pandemics involving the lower respiratory tract during the last two decades.

Children are not exempt from developing pneumonia from emerging pathogens like adults. The consequences, the likelihood of becoming infected and the prognosis will depend on the pathogen, incidence, and other risk factors. Over the past decades several microbiological agents, including viruses and bacteria that compromise the lower respiratory tract have emerged (Figure 1).

For the purposes of this review we have classified emerging pathogens into three categories: **True emerging** pathogens, to pathogens identified for the very first time affecting human population; **Re-emerging**, to known pathogens which circulation was controlled once, but they have reappeared or have developed significant antimicrobial resistance; and finally, those that we have called **old known with new presentations**, including pathogens that have always been present but in which new forms of respiratory involvement have been identified, other than those classically known so far.

In the first category we will discuss emerging new coronaviruses such as SARS-CoV-1, SARS-CoV-2, MERS-CoV, avian influenza and hantavirus (Andes virus). In the group of reemerging we will see the respiratory manifestations in children caused by measles, tuberculosis, bacteria with new developed antimicrobial resistances, new serotypes of post-vaccine pneumococcal; and in the old known with new behavior we will see rhinovirus and coronavirus (HCoV-NL63, HCoV-HKU1).

Traditional diagnostic methods, especially for viral respiratory pathogens, do not include many of these new agents so the possibility of identifying the etiology in episodes of low respiratory infections in children reaches 50% (1).

The objective of this review is to bring forward these respiratory pathogens, although with low frequency in children, which are certainly important to recognize to establish prognostics, appropriate therapy, and effective control measures. We do not include in this review certain pathogens, even though they were identified in the last 20 years, nowadays we know their behavior and it is possible to identify them with routine laboratory tests such as human metapneumovirus.

## True Emerging Pathogens

### Severe Acute Respiratory Syndrome Coronaviruses (SARS-CoV-1, MERS-CoV, SARS-CoV-2)

**Figure 2:**
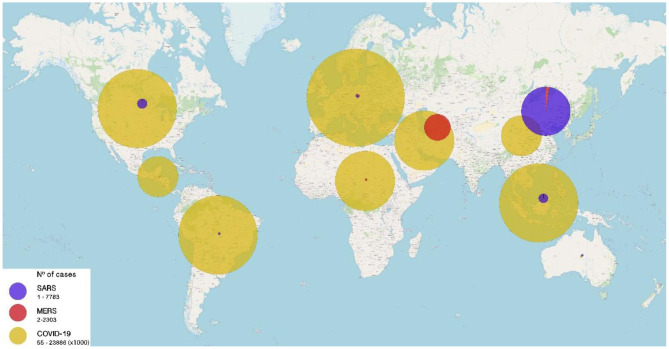
Number of cases of Severe Acute Respiratory Syndrome (SARS) caused by emerging coronaviruses worldwide from 2003 to 2020. SARS (purple), MERS (red), and COVID-19 (yellow) cases until December 21, 2020. Size of circles is proportional to the number of cases (range: 0–23,886,000 cases).

Coronaviruses are viruses with a single strand of RNA with positive polarity belonging to four genera: alpha, beta, gamma, and delta coronavirus. Human coronaviruses that have traditionally been responsible for the common cold or mild respiratory infections are HCOV-229E, HCoV-OC43, HCoV-NL63, and HCoV-HKU1. In the year 2003, the first coronavirus capable of producing a serious respiratory involvement with high mortality was identified, SARS-CoV-1, causing SARS and later, in the years to come MERS-CoV and recently in 2019 SARS-CoV-2, all belonging to beta coronaviruses and genetically closer to each other than to the traditional human coronaviruses (2), with zoonotic origin, probably bats. These coronaviruses are highly pathogenic and have high mortality when they infect humans (Figure 2).

#### Severe Respiratory Syndrome (SARS)

This syndrome with respiratory failure was identified in 2002 by outbreaks of severe pneumonia in southern China. In February 2003, the World Health Organization (WHO) reported an emerging disease with severe respiratory involvement. Quickly and in collaborative work, the causal agent was identified as a new coronavirus called SARS Coronavirus (3, 4), which was transferred from wild animal species to humans. The virus spread rapidly affecting 32 countries with more than 8,000 cases and 916 deaths between 2003 and 2004 with nearly 10% case fatality rate (CFR). Infection in children during the SARS outbreak was uncommon, ~6% of cases in the Hong Kong outbreak and less than 5% in China were under the age of 18 and most of them were infected within their household (5). No transmission was documented in schools. Overall mortality was around 15% where older adults and people with associated comorbidity were most at risk of death. In pediatric age, adolescents were the ones who had the highest risk of serious illness (6). The epidemic ended abruptly in July 2003 and just isolated cases were documented in humans until 2004.

##### Clinical Manifestations

After an incubation period of 5 to 10 days, the most common form of presentation in children was fever, dry cough, and runny nose. It differed from adults in whom the most common form of presentation was fever, cough, and dyspnea, associated with myalgias, headache, and shivers. However, adolescents over the age of 12 developed similar symptoms to adults, more severe lung involvement and hypoxia with oxygen requirement (7). No child had wheezing, lymphadenopathies, or visceromegaly (8). Laboratory tests highlighted the presence of lymphopenia in up to 64% of hospitalized children, with elevation of LDH and CPK. Lymphopenia as well as odynophagia and neutrophilia at admission, were identified as predictive factors of extensive lung involvement and severity (8, 9). Lymphopenia and thrombocytopenia were more common in children >12 years compared to <12 years who had rather reactive thrombocytosis. Radiological changes in the lungs of hospitalized patients were observed in 55% of children. These changes consisted mainly of consolidations (46%) and ground glass opacification (GGO), usually in peripheral locations. Multifocal consolidations were observed in 22%, followed by peribronchial thickening in 14%. Only 1 patient out of a total of 62 had pleural effusion and none showed interstitial pattern (10). In the long term, 34% of children in follow-up had both functional and radiological long-term abnormalities, however, the children were asymptomatic (9).

##### Diagnosis

SARS diagnosis was made in cohorts of children who met the clinical definition and had epidemiological links, using PCR. Only 48% had positive PCR taken within the first 8 days of symptoms and in 16% the virus was isolated by culture from upper airway samples. The sensitivity of diagnostic methods did not vary depending on the age or severity of the picture. Seroconversion occurred in nearly 100% of children studied within 21 days of onset of symptoms, being as early as 8 days. At 4 to 6 months of follow up most of the children still had positive antibodies although the titles had decreased by more than half (8).

##### Treatment

Almost all children received ribavirin as part of treatment and 85% of those hospitalized received steroids. Those over the age of 12 were also given methylprednisolone due to the highest frequency of most severe cases in this age group (8).

##### Prognosis

No deaths were described within the pediatric group with SARS, the risk of serious illness was much lower than in adult patients, except in those over 12 years of age where clinical and radiological manifestations and severity approach adults. In the long term, it is described that a quarter of children had muscle weakness and transient hair loss but with full recovery after a few months (8).

#### Middle East Respiratory Syndrome (MERS)

MERS-CoV was first isolated from a patient in Saudi Arabia in 2012. Since then and until September 2019, more than 2,500 cases and more than 860 deaths have been identified. The CFR of MERS globally reaches 34.4% (11) and has affected 27 countries, mainly in the Middle East (Figure 2). It is thought it has been transmitted from camels to humans. This virus continues to circulate to these days, with low frequency.

The incidence in children is much lower than in adults. During the epidemic, 38 pediatric cases have been reported and 3 deaths have occurred. In the Saudi Arabia's cohort with 1,791 cases, 30 correspond to pediatric cases (1.7%), and most of them were exposed to a household case (12).

##### Clinical Manifestations

After an incubation period of 5 to 7 days (2–14 days), the most common symptoms in adults are fever, dry cough, myalgias, shivers, arthralgia followed by dyspnoea. Asymptomatic or oligosymptomatic infection is estimated to reach 25–50% of infected individuals. Risk factors for serious illness include age >65, high fever, thrombocytopenia, and lymphopenia as well as the presence of comorbidities such as immunosuppression. In these cases, the risk of mortality is very high (13).

In children the infection is milder than in adults with cough being the predominant symptom (14). In the Saudi Arabia's pediatric cohort, including 30 cases, 3 deaths have occurred: a 9-month-old infant with nephrotic syndrome, a 2-year-old with cystic fibrosis, and a 15-year-old adolescent. The average age of children was 11 years (0–17 years) and 80% of them were over the age of 10. The infection was asymptomatic in 43% (10/23) of patients. Hospitalization reached 50% (12/24) of children. In this cohort the frequency of hospitalization and complications in children was significantly lower than in adults but there was not statistically significant difference in mortality. However, the fatality rate in children is about 8%, much lower than described for adults. In the Thabet's series, that accounts for 14 pediatric cases and 2 deaths, they observed that severe cases of MERS in children had the same presentation as in adults with multiple organ failure and acute renal failure (15). The radiological changes described in the 3 children with the most serious disease, were bilateral diffuse chest infiltrations.

##### Diagnosis

Like SARS, the diagnosis is based on upper respiratory PCR and later, by the presence of MERS-CoV antibodies (13). The duration of viral excretion in children, especially asymptomatic ones, that could represent an important source for community transmission is unknown (12). Facing a child with low respiratory infection and epidemiological criteria such as travel to the Middle East or contact with someone who has traveled to that area, MERS should be studied as a possible causal agent.

##### Treatment

Different antiviral therapies have been attempted in adults to treat MERS such as ribavirin, lopinavir-ritonavir, remdesivir, interferon alfa, convalescent plasma but none of them have been studied in randomized clinical trials. The use of corticosteroids does not appear to be indicated in MERS and may even delay viral clearance in critically ill patients (16). None of these therapies have been evaluated in children.

#### COVID 19

This emerging infection is caused by the latest identified coronavirus, SARS-CoV-2, and it is currently in progress. COVID 19 was decreed pandemic by WHO in March 2020 and since its inception in China at the end of 2019, has affected nearly every country and caused more than 100 million cases worldwide and more than 2.3 million deaths (Figure 2). COVID19 has become the most severe pandemic in the last 100 years comparable to Spanish influenza in 1918 (17).

SARS-CoV-2 would apparently have been passed on to humans from its natural reservoir, the bats, but the intermediary species has not been discovered yet. Since the description of the first cases in China in 2019, it has demonstrated its rapid adaptation to human beings, capable of infecting them easily with a high rate of person-to-person transmission. Its incubation period is on average 3 to 5 days with a range between 2 and 14 days. It is transmitted mainly by respiratory droplets and fomites. Aerosol transmission has not been completely ruled out and might play a role in certain situations such as enclosed environments and from severe patients under procedures such as intubation, ventilatory support, airway aspiration, etc.

Although it affects all age groups, children become infected much less frequently than adults and with less severity, corresponding to ~2% of the total COVID19 cases (18). Most children become infected within their households. Risk factors for a serious infection are age >65 years, presence of comorbidities such as diabetes, chronic kidney failure, hypertension, obesity, immunosuppression. Overall mortality is 2.2% reaching up to 25% mortality in risk groups (17, 19).

In hospitalized adults described in Richardson's series, 14% required intensive care (ICU) management, 12% mechanical ventilation, and 21% died of COVID19 (20). Risk factors for severity were male gender and the presence of comorbidities such as high blood pressure, obesity, and diabetes. The incidence of disease in children is much less common with 0.8 to 1.7% of all patients in different series. The median age in the different series varies between 7 and 11 years. There is no significant difference by sex (21, 22). Initially during the pandemic, it was thought that children did not become seriously ill, however more evidence has accumulated showing that children may require intensive care by ~2 to 6% (22). The age group at higher risk is infants <1 year, who may have severe disease in about 11% of them, compared to 3% of adolescents (22, 23). However, COVID19 overall mortality in children is very low, <0.1% compared to 34% of fatality in people over 80. The most described comorbidities in children are chronic lung diseases (but not asthma), cardiovascular disease, and immunosuppression.

##### Clinical Manifestations

Children with COVID19 have mainly fever (70%), which is shorter in duration compared to adults, and dry cough (60%). The presence of dyspnea is less common than in adults. In children, gastrointestinal symptoms are more frequent. Progression to severe illness was observed between 0.5 and 2% and required admission to ICU (21, 22).

The chest X ray in children was abnormal up to 49.1% even some of them being asymptomatic (23). Pulmonary involvement in CT scan described by Simoni et al. in a systematic review, which brought together 166 children, showed mostly bilateral infiltration, between 57 and 75%, and a peripheral distribution between 12.5 and 51.7% (24). The presence of GGO was the most common finding in children with pulmonary involvement, followed by the combination of GGO and consolidation according to different series (24, 25). Predominance of lower lobes and upper lobes has been described according to different series suggesting that in children there is not a clear pattern of pulmonary involvement location. The halo sign was frequently observed in children, between 12.5 and 50%, being a rare presentation in adults (25). Pleural effusion and interstitial involvement are rare findings, as is air bronchogram and interlobular thickening. Alterations in pulmonary CT have been observed in asymptomatic children or with mild manifestations. In newborn abnormal radiological findings were described in 48% but specific lesions were not as frequent as in older children; 4% had GGO, 20%, unilateral patchy infiltration and 12% bilateral involvement (23).

##### Complications

Children develop a severe lung infection with low frequency unlike adults. However, a complication, formerly known as pediatric inflammatory multisystem syndrome (PIMS) and later on named as Multisystem inflammatory Syndrome in Children (MIS-C), has been described in children who presented with shock and a hyper-inflammatory state like observed in Kawasaki disease and is temporarily associated with SARS-Cov-2 infection. The average age of presentation in a Chilean series including 27 children, was 6 years, 52% male. Fever was the most common symptom and gastrointestinal manifestations such as diarrhea, abdominal pain, and vomiting were present in 63% of cases. Around 67% of children had at least one Kawasaki disease criterion. Alterations in heart function were observed in 31% of children and 60% required ICU management (26). This syndrome tends to be later on the disease's stages. Frequently COVID PCR is negative and specific antibodies are positive in children with this type of complication.

##### Diagnosis

Diagnosis in the acute phase is made by nasopharyngeal PCR and by serology in the following days 80% of children negativize PCR in the upper respiratory tract between 1 and 15 days of onset of symptoms and 6% persist positive for up to 1 month (27).

##### Treatment

Treatment in children will depend on the severity of the disease. Only symptoms relief treatment is indicated for mild to moderate cases. In cases of severe respiratory involvement, supportive therapy with oxygen administration and ventilatory support when required is the main pillar. Clinical trials to study different treatments for children have not been done. Remdesivir and dexamethasone can be used in the case of severe respiratory symptoms. Hydroxychloroquine, lopinavir/ritonavir, ribavirin have not shown efficacy in the management of patients with severe COVID19 (27). For systemic hyperinflammatory syndrome, treatment like Kawasaki disease with intravenous immunoglobulin and steroids has been proposed (28).

### Avian Influenza

Two are the avian influenza viruses that have caused big outbreaks in humans in the last 15 years: influenza A H5N1 and influenza A H7N9. They share the same natural reservoir, wild and domestic birds, and have a low transmission capacity to humans but with serious lung compromise and high mortality.

The H5N1 avian influenza virus was first identified in birds by the death of geese in 1996 and the cases in humans were described for the first time in Guangdong, China in 1997. This outbreak was controlled with the slaughter of thousands of poultry. It re-emerges again in 2003 and since then, presents in seasonal waves during winter months being the last major wave of circulation in late 2014 and early 2015 (29). To date, 862 cases and 455 deaths with CFR close to 50% have been reported in 16 countries. The last human case diagnosed was in October 2020, in a 1-year-old infant in Laos (30). The H7N9 virus, second in importance in human cases, has a low pathogenicity in birds, first appeared in 2013, in Shanghai and since then 5 waves of human cases have occurred in China, the most intense being in 2017. In the latter wave the appearance of high pathogenic strains were documented. The last documented human case was in March 2019, an 81-year-old man in China. To date it accumulates 1568 laboratory-confirmed human cases and at least 613 deaths, with an overall mortality of 39% (31).

Birds are the main reservoir of influenza A virus. The H5N1 influenza virus emerged as a virus of high pathogenicity, that is, capable of producing great morbidity and mortality among birds, and when infects humans, which do not constitute its natural host, also causes serious lung involvement and high mortality. The H7N9 virus is a low pathogenic virus for birds, therefore it is very difficult to recognize it before cases in humans occur. The way of transmission is mainly from poultry or domestic birds to humans, but human-to-human transmission has also been identified in a much smaller proportion and sustained person to person transmission has not been described so far. The incubation period is longer than seasonal influenza being between 2 and 8 days but can reach up to 17 days. The influenza virus binds by its hemagglutinin molecule to the sialic acid receptor located in the airway epithelium. The human influenza virus has a higher affinity to the sialic acid-α 2,6 galactose receptor. The natural receptor of avian viruses is the sialic acid- α 2,3 galactose molecule found in the bird's airway. In humans, both receptors are present, the upper airway mainly contains the α 2,6 galactose while the lungs have α 2,3 galactose and sialic acid-α 2,6 galactose. Mutations in avian virus hemagglutinin promote the binding of avian virus to human influenza receptors in the airway. The H5N1 virus preferably binds to the α 2,3 galactose receptor, while H7N9 can bind both (32).

H5N1 infections are concentrated in a young population under the age of 40, while cases of H7N9 are grouped in persons over 50 years of age, probably due to the existence of oligosymptomatic or asymptomatic cases in children, which are not reported. There is a male gender predominance in H7N9 infection while in H5N1 there are no differences between sex. Different studies indicate the existence of a larger number of undetected mild cases of H7N9 due to a major proportion of asymptomatic cases. This suggests a more widespread genetic adaptability and higher susceptibility of humans to this virus and therefore with greater potential pandemic risk than H5N1. Influenza H5N1 has a higher frequency of clustered cases and a mortality somewhat higher than H7N9 (33, 34).

#### Clinical Manifestations

In a study series of 193 children with H5N1 the main symptom was fever. In children under 5 years of age, rhinorrhea was second in frequency after fever followed by vomiting and tachypnea. In older groups, the frequency of myalgia, odynophagia, and headache was higher. Tachypnea was equally common in all groups. Productive cough was more common in adolescents reaching 26%, than in younger children. Mortality was close to 50% and was higher in the adolescent group between 12 and 17 years old (80%) compared to children under 5 years of age whose mortality was 27% (35).

Upon admission to the hospital, which occurs on average on the fifth day of illness, all children showed abnormalities in chest X-ray. Initial changes were interstitial infiltrates that rapidly progressed to diffuse alveolar infiltrate with segmental distribution and air bronchograms mainly in the lower areas (36). No patients had pleural effusion, pneumothorax, or hilar lymphadenopathy. Patients with severe illness develop diffuse alveolar damage with progression in hours to adult respiratory distress syndrome. The laboratory highlights leukopenia with lymphopenia, thrombocytopenia, and transaminase elevation as a poor prognostic factor.

Clinical manifestations in children with H7N9 avian influenza are little described considering that most of the descriptions come from hospitalized patients. Because children are hospitalized less frequently than adults there is not much information about the clinical manifestation in them. Infection in children is most often mild or asymptomatic. The most common symptoms are high fever, cough followed by expectoration and dyspnea (37). Chest X-ray and CT Scan in several adults show alveolar involvement of GGO or multifocal consolidation, uni or bilateral, which progresses in extension rapidly in the first 2 weeks of onset of symptoms. CT compared to chest X-ray show greater compromise, becoming more sensitive in detecting lung damage (38).

#### Diagnosis

Diagnosis of avian influenza is made by specific PCR. Diagnostic tests of human influenza are not able to detect zoonotic influenza viruses so clinical suspicion based on clinical manifestation and epidemiology are relevant.

#### Treatment

The treatment of avian influenza is mainly based on antiviral drugs, neuraminidase inhibitors such as oseltamivir, peramivir, and zanamivir, which should be initiated immediately after clinical suspicion without diagnostic confirmation, given the severity of these infections (39). Oseltamivir is approved for use in children over 2 weeks of age, so it is the treatment of choice in children. However, it has been documented strains resistant to this drug considered the fundamental pillar of treatment (40). The duration of therapy, based on expert opinion, should be longer than for seasonal influenza, 10 days compared to 5 days, considering zoonotic influenza viruses have higher viral loads and longer viral replication. H5N1 and H7N9 viruses are adamantans resistant. Nitazoxanide, an antiviral that blocks the maturation of viral hemagglutinin, has shown *in vitro* to be effective against adamantine- and neuraminidase inhibitors resistant influenza viruses. It has synergistic effect with neuraminidase inhibitors *in vitro* (41). One study showed clinical efficacy in patients treated with 600 mg nitazoxanide twice daily for 5 days, in time to reduce symptomatology and in reducing the viral load of influenza, compared to placebo (42). Further studies are needed to confirm its effectiveness but could be an alternative to resistant strains in children as it is approved to be used over 1 year of age. The use of corticosteroids in patients with severe influenza H7N9 infection showed increased mortality at 30 and 60 days, so its use is not recommended (43).

### Hantavirus

Hantavirus infection can be divided into the one that occurs in the Old World and is mainly presented as a hemorrhagic syndrome with renal failure, and the one of the New World whose clinical manifestation is Cardiopulmonary syndrome (HCPS). It is this latest one that we will refer to in this article.

**Figure 3:**
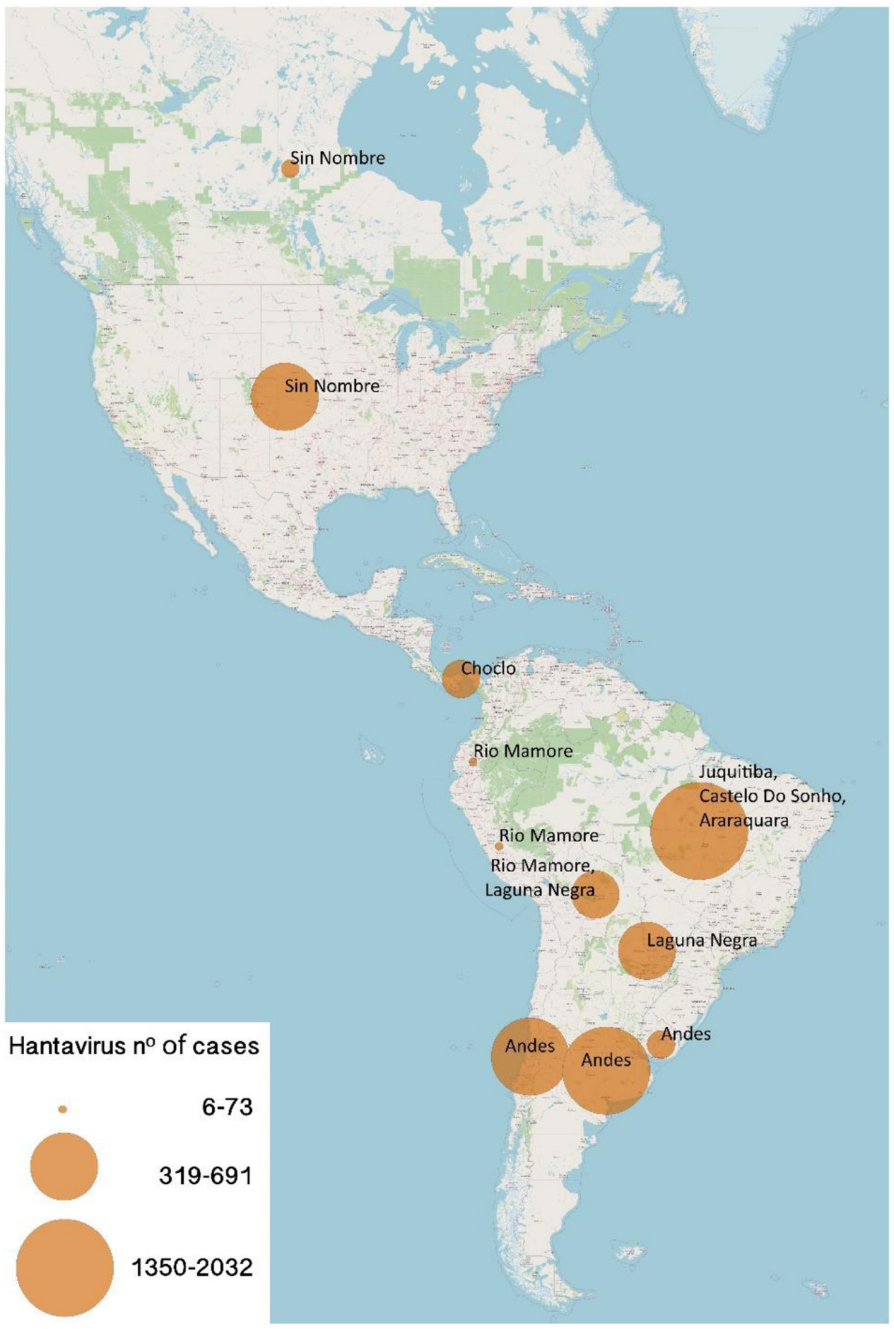
Numbers of New World Hantaviruses confirmed cases in the Americas. Size of circles is proportional to the number of cases (range 6–2032 cases).

Hantavirus infection was first documented in the United States in 1993 when cases of people with acute respiratory failure and shock appeared. The identified virus was called Sin Nombre virus (SNV). Subsequently, the same clinical entity was recognized in Argentina and Brazil in 1993 and 1994 respectively. Later, Chile diagnosed the first case in 1995 (Figure 3). Although hantavirus infection is currently endemic in many countries of the Americas, it is considered an emerging virus, capable of causing severe respiratory involvement, from which we are still learning its clinical course and treatment, and which must be suspected in patients with severe lung compromise associated with cardiogenic shock.

HCPS is produced in the Americas by different types of hantaviruses, in the USA is SNV, Argentina and Chile Andes virus (ANDV), in Brazil Laguna Negra virus or Castelo Do Sonhos virus, among others. The natural reservoir are wild rodents, which are specific to each type of hantaviruses and it is transmitted by inhalation of aerosolized viral particles from rodent feces and urine. Person to person transmission in ANDV has been confirmed and evidence still under investigation suggests possible transmission through breast milk (44–47). Children have the same risk factors of becoming infected as adults such as exposure to contaminated areas by wild rodent fluids. However, for ANDV the presence of a household member with hantavirus infection is also a risk factor given its rare but confirmed person to person transmission of this viral type.

Given the transmission through inhalation of viral particles from the feces or urine of wild rodents, most cases occur in young men during work activities in rural areas and 25% are associated with recreational activities. Other identified risk factors for infection include caring, sexual partner and sleeping with an infected person.

Mortality varies between hantaviruses. SNV, ANDV, Araraquara, and Juquitiba are responsible for the most severe manifestations with case fatality rates ranging from 25 to 40%. Hantaviruses in Panama (Choclo virus) and Paraguay (Laguna Negra) have a lower CFR of 10 and 15% respectively, causing milder infections (48).

In the Chilean series of cases, including 997 cases of ANDV, from the first cases identified in 1995 to 2016, children account for 18.6% of cases and the CFR of this group <15 years is 31%, lower than people of 45 to 59 years who have a CFR of 42.8% (49). Approximately 60 cases are reported on average annually in Chile, with an average incidence of 0.3/100,000 inhabitants in the last 20 years, with most cases concentrated during the summer months (50).

#### Clinical Manifestations

After an incubation period, which for ANDV virus is between 7 and 39 days with a median of 19 days, and for SNV the maximum incubation period can be up to 17 days, the patient complains of non-specific symptoms such as fever, headache, myalgias, arthralgias. After this prodromal period, the patient develops cardiopulmonary syndrome about 5 days after onset of symptoms, starting with respiratory symptoms such as cough and dyspnea and progressing rapidly to acute non-cardiogenic pulmonary edema caused by increase of capillary leakage associated to cardiogenic shock, which is the main cause of death (48). In cases of human-to-human transmission the incubation period ranges from 12 to 29 days (44–46). The median between onset of symptoms and death is 5 days in the Reyes series (49). In a study of 32 infected Brazilian children showed similar clinical manifestations to adults identifying the two phases, prodromal, with non-specific symptoms and the cardiopulmonary syndrome phase, where cough (46.9%) and dyspnea (59.4%) appears; 59.4% of these children were hospitalized and the CFR was 34.4%, like was described by Reyes (51). In countries such as Barbados where hantavirus infection is milder, the infection in children can occur as a non-specific febrile syndrome mistaken for arbovirus infections such as dengue (52).

The laboratory findings include thrombocytopenia in 94.7%, hemoconcentration in 63.1%, left shift leukocytosis in 47.3%, and atypical lymphocytes in 26.3% (51).

**Figure 4:**
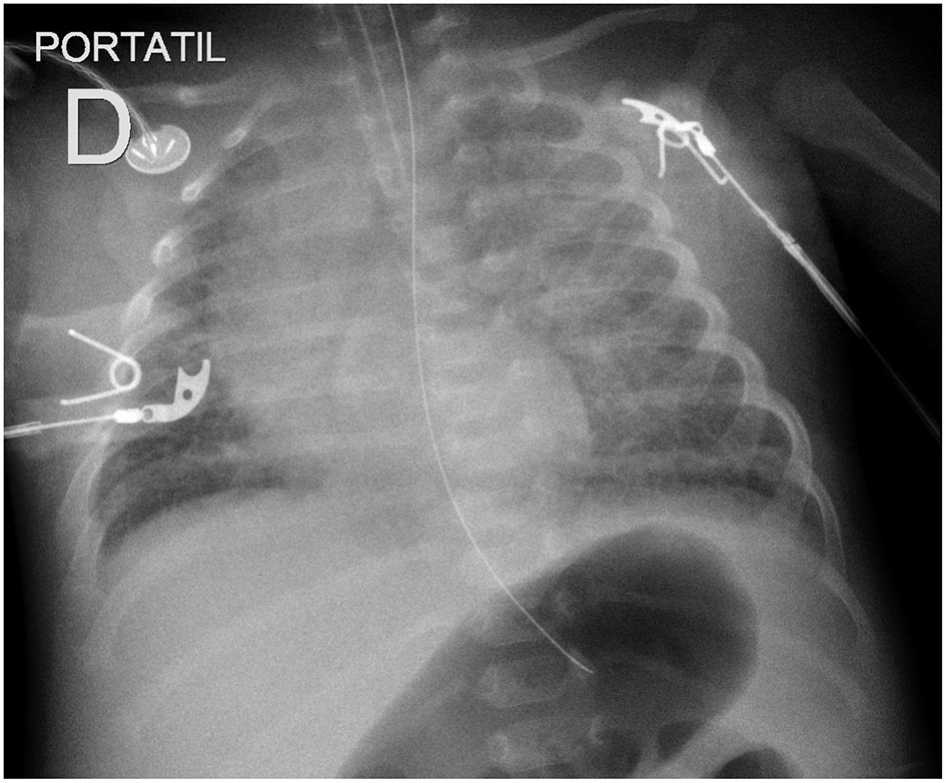
Chest X-ray showing diffuse interstitial and alveolar infiltrates in a newborn with Hantavirus infection.

Typical radiological findings in children include interstitial infiltrate in the initial stage followed by alveolar infiltrate, bilateral, compatible with pulmonary edema. Pleural effusion may also be observed during the cardiorespiratory phase by increasing capillary permeability (Figure 4).

#### Diagnosis

The method of choice is the determination of hantavirus-specific IgM and IgG antibodies, which are present at the time of onset of symptoms of cardiopulmonary syndrome phase. There is a cross-reaction between the different hantaviruses so it is not possible to determine the type by antibodies. The sensitivity of serology decreases during the prodromal period, so a negative result at this stage does not rule out the diagnosis of hantavirus infection.

PCR can detect viremia 5 to 15 days before seroconversion and onset of symptoms of HCPS (44). For this reason, it is the method of choice for the monitoring of close contacts of confirmed cases, to be able to diagnose infection early on, even before symptoms appear, to start early with support measures to infected contacts. It can also be used for the diagnosis of patients in its acute phase due to its higher sensitivity, although its availability is low.

#### Treatment

The main treatment is the relief of symptoms and the use of support measures in a critically ill patient, such as oxygen therapy, use of vasoactive drugs, different types of ventilatory support including the use of ECMO in the most severe cases.

There is no specific antiviral therapy as *in vitro* efficacy of antivirals such as ribavirin has not been demonstrated *in vivo*. The use of convalescent plasma was shown to decrease CFR significantly, although it did not reach statistical significance (53). This is the standard therapy today in Chile for hospitalized patients with ANDV infection. Corticosteroids are not beneficial and should not be used to treat hantavirus cardiopulmonary syndrome.

Tables 1 and 2 are a summary and show a comparison between these true emerging diseases.

**Table 1:** Comparison of epidemiological, clinical, and radiological features between major emerging pneumonia in children.

**Disease**	**Epidemiology**	**Clinical features**	**Radiology**
MERS	- Middle East - <2% cases are children - 80% cases are > 10 yo	- Less severe than adults - Main symptom is cough	Bilateral diffuse infiltrations
COVID19	- <2% cases are children - Median age 7–11 yo - Severe disease more frequent in <1 yo, respiratory comorbidities and immunosuppresion	- Mild disease - Main symptoms are fever and dry cough - MIS-C is the main complication	- Bilateral infiltration - Bilateral ground-glass opacities and consolidation
Avian influenza			
H5N1	- Present in China, South East Asia - Affects mainly population lesss 40 yo	- Severe disease in children - Main symptoms are fever, rhinorrhea, vomiting, tachypnea - Leukopenia, lymphopenia, thromocytopenia	Diffuse alveolar infiltrate, air bronchograms in lower areas
H7N9	- Present in China - Greater transmission in children compared to H5N1	- High proportion of asymptomatic and mild disease. - Fever, cough	Bilateral ground-glass opacities and consolidation
Hanta cardio pulmonary syndrome (HCPS)	- The Americas - ANDV <20% cases in children	- Main symptoms are fever, mialgia, cough - Severity varies regarding to type of Hantavirus - Thromocytopenia, hemoconcentration - HCPS is the main complication	- Interstitial infiltrate in the initial stage. - Followed by alveolar infiltrate, bilateral, compatible with pulmonary edema.

**Table 2:** Comparison of diagnosis, treatment and prognosis between major emerging pneumonia in children.

**Disease**	**Diagnosis**	**Treatment**	**Prognosis**
MERS	- PCR and serology	- Antivirals - Convalescent plasma - Not steroids	- Less hospitalization - CFR 8% in children with comorbidities
COVID19	- PCR in early stage - Serology after the first week	- Symptoms relief - Supportive therapy - Remdesivir - DexamethasoneMIS-C: - IV Immunoglobuline plus steroids	−2–6% hospitalization - CFR <0.1%
Avian influeza			
H5N1	- PCR	- Oseltamivir	- Overall CFR 50% - 80% in adolescents - 27% <5 yo
H7N9	- PCR	- Oseltamivir - Not steroids	- CFR <3%
Hanta cardio pulmonary syndrome(HCPS)	- Hantavirus specific IgM-IgG	- Support measures - Convalescent plasma - Not steroids	- ANDV, Laguna Negra, Castelo dos Sonhos, Sin Nombre CFR 31%

## Re-Emerging Pathogens

### Measles

Measles is a highly contagious viral infectious disease, with an estimated R0 of 12–18, which could be higher according to recent literature (54). The CFR reaches 5% but can rise to 30% when health service is not accessible or under humanitarian crises (55). Since the global introduction of the measles vaccine in the 1960s, the burden of the disease has decreased considerably worldwide. Despite the vaccine having an excellent efficacy and immunogenicity, some outbreaks continue to be seen around the world. However, during the last few years, these outbreaks have become more frequent, particularly in Africa and some European countries (56, 57). The patients were children and adolescents unvaccinated or with incomplete vaccination. The main risk factor for an outbreak is a low measle vaccination coverage, leaving a larger percentage of the population susceptible to the infection. Lack of vaccination can be due to a low coverage in an entire country, but it also occurs in countries with high vaccination coverage, which have groups of people with no access to healthcare, religious or philosophical beliefs who choose not to vaccinate their children (56–58). These outbreaks are controlled with emergency vaccination campaigns, educational, political, and technical assessments (59). To achieve measles elimination and to obtain herd immunity is essential to reach a 95% of vaccination coverage worldwide (60).

#### Clinical Manifestations

Measles is transmitted by droplet but also by small aerosolized particles favorizing contagiousness (61). After 7–14 days of incubation, the prodromal period appears characterized by high fever along with conjunctivitis, coryza and cough, which lasts 2–4 days. One or two days later, an oral mucosa involvement is observed with small white lesions at the level of the first molar known as Koplik spots, pathognomonic of measles. Then an erythematous morbilliform exanthema begins on the face and then spreads to the trunk and extremities. Symptoms usually last 7 days, and sometimes a fine scaling follows the rash (55). Measles can complicate with bacterial infection as otitis media or tracheitis, but can compromise any other organ presenting diarrhea, hepatitis, myocarditis, or less frequently central nervous system involvement (subacute sclerosing panencephalitis) (61).

Respiratory compromise, particularly pneumonia, is the most common severe complication and is responsible for hospitalization and death among children with measles (62). Pneumonia can be produced by other viral infections as adenovirus or rhinovirus, bacterial secondary infection, or measles alone (63, 64). Early in the course of the illness, after 2–7 days of fever and presence of rash, patients can evolve with dry cough and dyspnea. Young children characteristically present with bronchiolitis. In a recent measles epidemic developed in Italy, 30 (17%) young adults hospitalized with measles presented pneumonia, all of them unvaccinated (64). Five of them developed severe respiratory failure and two died. Having pneumonia was associated with thrombocytopenia and leukopenia. Most frequent findings in chest CT were bilateral lesions as centrilobular nodules and ground-glass opacity.

#### Treatment

Management of measles pneumonia is based on supportive measure. The role of antibiotics is still controversial. A review suggests, with poor evidence, a benefit of using antibiotics to prevent bacterial superinfection such as pneumonia or otitis media (65). Because cough is often dry, difficult the possibility to obtain bacterial culture and empiric antibiotics frequently are added in severe pneumonia or when secondary bacterial pneumonia is suspected by radiology (62, 64). Regarding vitamin A, there is still lacking evidence to prove it benefice reducing pneumonia associated mortality in children older than 2 years old, but the WHO recommend it use even in well-nourished children (66, 67). There is not sufficient evidence to support zinc supplementation as it has not demonstrated any effect on clinical outcome of children with measles (68).

### Pulmonary Tuberculosis

Tuberculosis (TB) is an infectious disease caused by *Mycobacteria tuberculosis* that has had a major impact on public health along the history of humanity. However, with hygiene improvement, social and economic development, production of appropriate drugs therapies and preventive measures, the incidence of this disease has significantly decreased, particularly in high-income countries (69). On the contrary, in low and middle-income countries, tuberculosis continues to be a leading cause of morbidity and mortality, with 44% of TB cases in South-East Asia, 25% in Sub-Saharan Africa, 18% in Western Pacific, and 8.2% in Eastern Mediterranean (70, 71). In 2019, the WHO estimated that 10 million people were ill with TB, with 1.2 million deaths among HIV-negative individuals and 208.000 among HIV-infected subjects (70). Regarding age distribution, 12% of total cases, and 16% of death from TB were in children under 15 years old, without gender predominance (70).

Few decades ago, tuberculosis re-emerged due to multifactorial causes, increasing its incidence in some countries and slowing its decline in others. The HIV epidemic left this population susceptible to mycobacterial infection, which increased the incidence of TB particularly in Sub-Saharan Africa and South East Asia (70, 72). Tuberculosis is the major cause of morbidity and mortality in HIV infected individuals, being the most common cause of hospital admission in this population (72). On the other hand, the increase in the mobility of population allowed the disease to reappear in places where incidence was low, as in Western Europe (73). Frequently, migrants are exposed to over-crowded facilities, poor hygiene conditions and inadequate access to health care systems increasing their risk of infection and appearance of multidrug resistant TB (MDR-TB). The appearance of these resistant mycobacteria has made the treatment and eradication of the disease difficult. The WHO estimates that 3.3% of new TB cases and 18% of cases already treated were MDR-TB, and the countries with the highest prevalence are India, China, and Russian Federation (70). Among the pediatric population is estimated that 3% of infected children have MDR-TB (74). Finally, the development of new therapies such as anti-TNF, which leave the host highly susceptible to mycobacterial infection, have reflected a reappearance of this infection, particularly in high-income countries (75).

#### Clinical Manifestations

Tuberculosis infection in children is most frequently asymptomatic. Younger children are at a highest risk to present a symptomatic disease (76). When symptoms occur, they usually begin 1 to 6 months after primary infection but can appear several months after. Non-specific symptoms are often seen such as failure to thrive, and less frequently fever, night sweats or chills. Intrathoracic involvement is the most common manifestation, with enlarged lymphadenopathy or pulmonary lesions (77). Persistent cough, wheezing, or dyspnea can be present and be confused with viral or bacterial pneumonia. Pulmonary involvement varies with age; adolescents can present with typically childhood manifestation but also with a cavitary phenotype as seen in adults (77). Children under 5 years old are at a higher risk to present a disseminated disease with compromise of other organs, such as lymphadenopathies, CNS and osteoarticular, among others.

Radiological findings in pulmonary TB are non-specific, but intrathoracic lymphadenopathy, airway compression, air-space disease, and less often military nodules and cavitation (78). CT is more sensitive to identify lymphadenopathy and parenchymal compromise. Further research should evaluate the role of magnetic resonance imaging and ultrasound for the diagnostic of intrathoracic TB.

#### Diagnosis

As clinical and radiologic manifestation are poorly specific, pulmonary TB diagnosis in children is challenging. Moreover, children usually have a low bacillary load, which, added to the low sensitivity of diagnostic techniques and the difficulty in sampling, complicates the identification of mycobacteria. A recent meta-analysis demonstrates a lower positivity in smears from children, particularly those younger than 4 years old, compared to adults (79). Mycobacterial culture is the gold-standard for TB diagnosis but is a time-consuming technique and its sensitivity can be as low as 7–40% in children (77).

A recent meta-analysis showed that molecular technique as Xpert MTB/RIF performed in expectorated, induced sputum or gastric lavage has a higher sensitivity compared to microscopy. However, this rapid confirmatory method is still less sensitive than the culture, so a negative test does not rule out the infection (80). To improve the sensitivity of this method, repeated sampling is recommended in the pediatric population (77). Regarding screening tests to evaluate evidence of exposure to mycobacteria, IGRAs have a high specificity, particularly in BCG-vaccinated children, and sensitivity above 90% in children older than 2 years old. Nonetheless, a negative result in a symptomatic child never rules out the infection (81).

#### Treatment

For the management of pulmonary TB in children, the WHO recommends a regimen of 2 months of three drugs (isoniazid, pyrazinamide, and rifampin) and 4 months of two drugs (isoniazid and rifampin), with exception of extensive pulmonary disease, living in settings with high prevalence of HIV or isoniazid resistance or in HIV-infected children (82). In these scenarios, a regimen of four drugs (isoniazid, pyrazinamide, rifampin, and ethambutol) and two drugs for another 4 months (isoniazid and rifampin) is recommended. When MDR-TB is suspected, it is essential to confirm the diagnosis, with culture and rapid drugs susceptibility testing as molecular tests (Xpert MTB/RIF). Regimens of 4 to 5 drugs (as fluoroquinolones, linezolid, clofazimine, ethambutol, among others) for 9 to 18 months or shorter regimens including an intravenous drug are recommended (74).

### Bacteria With New Developed Antimicrobial Resistances

#### Community-Acquired Methicillin-Resistant Staphylococcus Aureus (CA-MRSA)

##### Pathophysiology

*S. aureus* has many virulence factors that help it to instigate colonization, evade host-immune responses, cause tissue injury, and disseminate to other organs. In establishing infection, *S. aureus* expresses surface proteins that mediate adherence and impair local defenses, while later in the infection secreted exotoxins disrupt epithelial barriers and immune cell function responses, thereby facilitating tissue invasion (83).

Although it has long been recognized as an important cause of necrotizing pneumonia (NP), the interest in this pathogen was renewed by recent studies linking strains expressing the virulence factor, Panton-Valentine leukocidin (PVL), with severe forms of this disease in previously healthy children and adults (84–86). In many cases these PVL-producing isolates were also MRSA strains. PVL is a pore-forming exotoxin, which activates and then destroys immune cells, such as neutrophils, potentially releasing damaging proteases into the surrounding tissues (87, 88). Of concern, a multi-center French study (89) involving 50 cases of necrotizing pneumonia caused by PVL-producing strains of *S. aureus* in children and adults aged between 1 month to 78 years, reported a CFR of 56%. Factors associated with mortality were hemoptysis, erythematous rash within 24 h of admission and peripheral blood leukopenia <3.0 × 10^6^/L. However, this was a non-comparative study, and it is therefore difficult to infer whether PVL contributed to pathogenicity.

Indeed, whether PVL itself is responsible for the pathological changes seen in NP is controversial. In part, this is because PVL has a strong cell and species specificity, behaving differently in various cell cultures and experimental models. For example, neutrophils from humans and rabbits are very sensitive to the effects of PVL *in vitro*, while those from monkeys and mice are highly resistant (89). Moreover, while a systematic review and meta-analysis found a strong association between PVL producing strains of *S. aureus* and skin and soft tissue infections, no such association was seen for invasive infections, including pneumonia (90). However, this review include only a small study in children from China, which compared cases of methicillin-resistant (MRSA) community acquired pneumonia (CAP) and no significant differences in the proportions of PVL-positive (3/22) and -negative (3/33) strains progressing to NP were found (91). Similarly, linking necrotizing pneumonia with MRSA is also controversial. Many of the observational studies reporting an association between invasive disease and MRSA are from the US, where the PVL-producing USA300 MRSA clone predominates, while in Europe, Australia and elsewhere, there are many different MRSA strains circulating (92). Furthermore, a recent case-control study of 133 French children and adults with PVL-positive strains of *S. aureus* necrotizing pneumonia, found no evidence for increased clinical severity in those with MRSA infections (93). Consequently, there are substantial gaps in our knowledge concerning the pathogenesis of *S. aureus* necrotizing pneumonia and it is likely that other cytotoxins may play an important role. Indeed, attention has been focused recently on other pore-forming toxins including alpha-hemolysin (or α-toxin), with its proposed mechanisms of action including activating the NLRP3 inflammasome, resulting in severe alveolar necrosis, and inducing platelet-neutrophil aggregation, which leads to further tissue destruction (94).

Community-associated MRSA (CA-MRSA) and healthcare-associated MRSA (HA-MRSA) isolates are distinct entities on a molecular level although the terminology is loosely used in the literature. MRSA infections that arise outside the hospital setting are conventionally labelled as “community-acquired” or “community-onset.” These can be caused by both the newly emerged CA-MRSA strains as well as HA-MRSA strains that have “escaped” from hospital via colonized patients. Conversely, in areas where the prevalence of CA-MRSA is high, nosocomial infections are increasingly due to CA-MRSA (95).

Infections due to CA-MRSA were first reported in the early 1980s in aboriginal communities living in the Kimberley region of Western Australia (96, 97). The Australian Group on Antimicrobial Resistance in its 2006 *Staphylococcus aureus* Program (98) reported an 87% increase in the number of community-onset *S. aureus* infections due to MRSA in Australia. CA-MRSA clones accounted for 56.7% of all MRSA and 8.8% of all *S. aureus* isolated. Multilocus sequence type 93-MRSA-IV (Queensland strain) was the most frequently isolated CA-MRSA clone. The CA-MRSA as a cause of CAP in children and healthy adults is reported more frequently, mainly in case reports and small case series (99, 100).

##### Clinical Manifestation

The combined actions of many virulence factors enable *Staphylococcus aureus* to cause disease (101, 102). Depending on these factors and on the immune status of the host, *S. aureus* can cause diseases ranging from superficial skin infections to deep infections such as osteomyelitis, septic shock, and necrotizing pneumonia. Staphylococcal necrotizing pneumonia can affect young, immunocompetent patients (103). This disease, characterized by leukopenia, hemoptysis, and extensive necrosis of the lung tissue, is caused by *S. aureus* strains that produce PVL (104).

##### Treatment

Recommended therapy for CA-MRSA pneumonia includes vancomycin, linezolid, or clindamycin for 7–21 days. For necrotizing pneumonia, clindamycin with rifampin, vancomycin with rifampin, linezolid with rifampin or vancomycin with clindamycin have been successful in longer duration completing up to 4 weeks of treatment.

#### Mycoplasma Pneumoniae

*M. pneumoniae* is a common pathogen that causes CAP in children. The proportion of pneumonia caused by *M. pneumoniae* in different studies ranged from 20 to 40% (105). Increasing numbers of refractory or severe *M. pneumoniae* pneumonia cases have been reported worldwide, especially in Asia (106). Previous studies have shown that refractory *M. pneumoniae* pneumonia is associated with prolonged fever, high levels of C-reactive protein, airway hypersecretion, and consolidation on chest imaging (107). It has been confirmed that the excessive immune response of the host plays an important role in the development of refractory *M. pneumoniae* pneumonia (108). In this context, corticosteroids have been suggested as an immunomodulator for downregulating the overactive host immune reaction. Previous research confirmed that coinfection with viruses and bacteria led to more severe disease in children with refractory *M. pneumoniae* pneumonia (109). In general, viral coinfection rates in children with *M. pneumoniae* pneumonia ranged from 10 to 30% (110). A recent study done in Shanghai showed 56% coinfection and infection by drug-resistant *M. pneumoniae*. The viral coinfection was more common in patients younger than 3 years old. Adenovirus coinfection and drug-resistant *M. pneumoniae* infection occurred significantly more commonly in patients with refractory *M. pneumoniae* pneumonia (111).

Macrolide-resistant *M. pneumoniae* infection may also play an important role in the occurrence and development of refractory *M. pneumoniae* pneumonia (112). Overuse of macrolides may contribute to macrolide resistance, and thereafter, an increase in macrolide-resistant *M. pneumoniae* pneumonia. Mutations at position 2063 or 2064 domain V in the 23S rRNA gene are related to macrolide resistance (113). Some Chinese series reported a rate of drug-resistant *M. pneumoniae* around 70 to 90%; however, macrolide resistance is less common in the US and European countries, where the macrolide-resistant *M. pneumoniae* prevalence is below 30% (114). Maybe that relatively high mutation rate in China is probably related to excessive exposure to macrolides for respiratory infections in outpatients, especially in children. A study from Japan reported that the macrolide-resistance rate decreased to 59.3% in 2014 and 43.6% in 2015 from the highest macrolide-resistance rate of 81.6% in 2012 (115), may be attributed to the decrease in the use of oral macrolides. In Japan, tosufloxacin was approved for pediatric used in macrolide-resistant M. pneumoniae (MRMP) pneumonia; however minocycline or doxycycline were significantly more effective in achieving defervescence within 24 h and in decreasing numbers of *M. pneumoniae* DNA copies 3 days after initiation (116).

Macrolide-resistant *M. pneumoniae* pneumonia shows persistent fever and/or no radiological regression to macrolide antibiotics and may even progress to severe and complicated pneumonia.

##### Treatment

In children with drug-resistant *M. pneumoniae* pneumonia, tetracyclines (doxycycline, minocycline) have shown excellent efficacy (117). Because of adverse reactions, tetracyclines are contraindicated in pregnant women and children under 8 years old. However, previous studies showed that short and limited courses of treatment (less than 6 courses, 6 days per course) caused insignificant tooth discoloration in children under 5 years old. Delayed effective antimicrobial treatment is associated with prolonged and/or more severe disease. Thus, the appropriate prescription of antibiotics, as well as the rapid and accurate diagnosis of *M. pneumonia*e pneumonia is important.

#### Gram-Negative Multiresistant Bacterias

In this article, we will review the main resistant gram negative pathogens causing pneumonia: *Acinetobacter baumannii, Pseudomonas aeruginosa*, and *Stenotrophomonas maltophila*. All of them are responsible mainly for nosocomial pneumonia.

##### Acinetobacter Baumannii

Acinetobacter baumannii is a major cause of nosocomial pneumonia in certain geographic areas affecting mainly debilitated patients, with prolonged hospitalization and broad-spectrum antimicrobials treatments (118). *A. baumannii* is mainly transmitted via hands of healthcare workers or fomites (119). However, the airborne route also plays an important role in spreading *A. baumannii*. Inappropriate empirical treatment has clearly been associated with increased mortality in *A. baumannii* pneumonia. *A. baumannii* spreads rapidly and possesses an extraordinary capability to develop resistance to almost all antibiotics (120). *A. baumannii* has innate resistance mechanisms against multiple antimicrobials on its core genome. Moreover, this pathogen easily acquires new resistances by diverse mobile elements. These include enzymatic inactivation, alteration of bacterial targets, permeability barriers, or active efflux pumps. Carbapenems may not be considered the treatment of choice in areas with high rates of carbapenem-resistant *A. baumannii*. Nowadays, polymyxins are the antimicrobials with the greatest level of activity *in-vitro*. Colistin is the antimicrobial most widely used although polymyxin B is associated with less renal toxicity. However, lung concentrations of polymyxins are suboptimal in a substantial proportion of patients. Regarding nebulized antibiotics, it seems reasonable to use in patients who are non-responsive to systemic antibiotics or *A. baumannii* isolates with colistin minimum inhibitory concentrations close to the susceptibility breakpoints. Cefiderocol, a novel cephalosporin active against *A. baumannii*, may represent an attractive therapeutic option if ongoing clinical trials confirm preliminary results (118). However, well-designed, randomized controlled trials must be conducted to comprehensively evaluate the effectiveness and safety of nebulized antibiotics for the treatment of *A. baumannii* pneumonia.

##### Pseudomonas Aeruginosa

Nosocomial pneumonia due to *P. aeruginosa* is associated with considerable morbidity, prolonged hospitalization, increased costs, and mortality. *P. aeruginosa* is one of the few pathogens independently associated with increased mortality among patients with sepsis or pneumonia in the ICU setting (121). The mortality associated with *P. aeruginosa* pneumonia is further increased when inappropriate initial antibiotic therapy is prescribed, usually due to the presence of multidrug-resistant (MDR) pathogens. The overall impact of *P. aeruginosa* pneumonia on clinical outcomes and healthcare costs underscores the importance of this nosocomial infection. In recent study assessing the multinational burden and specific risk factors associated with *P. aeruginosa*-CAP, 3193 patients were enrolled in 54 countries with confirmed diagnosis of CAP who underwent microbiological testing at admission. The prevalence of *P. aeruginosa* and antibiotic-resistant *P. aeruginosa*-CAP was 4.2 and 2.0%, respectively. The rate of *P. aeruginosa* CAP in patients with prior infection/colonization due to *P. aeruginosa* and at least one of the three independently associated chronic lung diseases (tracheostomy, bronchiectasis and/or very severe chronic obstructive pulmonary disease) was 67%. In contrast, the rate of *P. aeruginosa*-CAP was 2% in patients without prior *P. aeruginosa* infection/colonization and none of the selected chronic lung diseases. The multinational prevalence of *P. aeruginosa*-CAP was low (122). A recent retrospective cohort study in adults showed that almost 31% of patients with *P. aeruginosa* pneumonia were infected with MDR strains. In multivariable analyses, independent predictors of MDR *P. aeruginosa* included age, diabetes mellitus and ICU admission. MDR strains, heart failure, increasing age, mechanical ventilation, and bacteremia were independently associated with in-hospital mortality (123).

Among pediatric patients, this organism is prevalent in pediatric intensive care units (PICU), and its incidence as a nosocomial lung infection has doubled over the last three decades (124). *P. aeruginosa* is intrinsically resistant to several antimicrobial agents, and it can acquire resistance to many others. In recent years, the frequency of multidrug-resistant MDR strains of *P. aeruginosa* is increasing, especially in nosocomial infections and PICU-acquired infections (125), and these infections increase mortality, morbidity, and hospital costs. The mortality of children with *P. aeruginosa* infection ranges from 20 to 50% in Chinese reports (126) and 33 to 61% (127) in other populations.

Among the new antibiotics approved for *P. aeruginosa* are: ceftolozane/tazobactam for extended spectrum β-lactamases (ESBL) *P. aeruginosa* and meropenem/vaborbactam for AmpC β-lactamases *P. aeruginosa* (128).

##### Stenotrophomonas Maltophilia

*Stenotrophomonas maltophilia* is a commensal and an emerging pathogen earlier noted in broad-spectrum life-threatening infections among the vulnerable, but more recently as a pathogen in immunocompetent individuals (129). In addition, *S. maltophilia* has emerged as an important pathogen that induces nosocomial infections (130). *S. maltophilia* is a non-fermentative, gram-negative bacilli and causes severe infectious diseases, such as pneumonia, bacteremia, skin and soft-tissue infection, urinary tract infection, and meningitis (131). Recently, the frequency of infection reported worldwide is quite alarming. *S. maltophilia* accounts for about 3.7% (*n* = 10,000) in hospital discharges (132). A recovery rate of 3.3% in *S. maltophilia* infections was reported in the US (124). The mortality rate due to *S. maltophilia* infection was 36.6% found in a large study (133) and 25–51% in a multi-center study (134). Pathogenesis is by colonization, rather than infection, which is often accompanied by tissue invasion (135). The most important risk factors for *S. maltophilia* infection in neonates and infants are invasive procedures; previous exposure to antibiotics, such as carbapenem and aminoglycoside; and prolonged hospitalization (136). The duration of hospitalization before the onset of the *Stenotrophomonas* clinical symptoms is an important factor in nosocomial infection. The duration of hospitalization before the onset of *S. maltophilia* bacteremia ranges from 11.5 to 24 days (137, 138). Neonate and infants have low immunity, and often have severe and uncontrollable symptoms after infection. *S. maltophilia* is intrinsically resistant to many antibiotics, including carbapenems and aminoglycosides which are used empirically for nosocomial infection (130). Therefore, early identification and appropriate treatment are important.

##### Treatment

*S.maltophilia* is intrinsically resistant to Beta-lactams (penicillins, cepahosporins, aztreonam, and carbapenems) due to chromosomal metallo-beta lactamase and extended-spectrum beta lactamases (ESBLs) production.

Treatment of choice includes trimethoprim-sulfamethoxazole, levofloxacin, minocycline and ceftazidime. Therapy should be guided by *in vitro* susceptibility results.

### Other Re-emerging Bacteria

#### Streptococcus Pneumoniae

WHO reported that pneumonia accounts for 16% of all deaths of children under 5 years old, killing around 1 million children in 2015, with the most common cause of bacterial pneumonia being *S. pneumoniae* (139). *S. pneumoniae* was estimated to be responsible for 341,029 deaths of children younger than 5 years in 195 countries in 2016 (140). Serotype 1 has been predominantly responsible for empyema in a study from UK, although, there is no information about the most frequent serotypes found in pneumonia in this country (141). In a recent review of 197 bacterial and fungal pathogens detected in single case reports and case series, *S. pneumoniae* accounts for 116 (59%) of necrotizing pneumonia (142).

Pneumococci possess multiple virulence factors (143), including its polysaccharide capsule, cell surface proteins, the cell wall, and pneumolysin, a pore-forming toxin (144). Of these, the most important is the polysaccharide capsule, of which there are at least 98 different serotypes, each capable of shielding the organism from the immune system (145). Individual serotypes vary in their capacity to colonize, cause local or invasive disease, and express antibiotic resistance genes (143). Serotypes also vary geographically and change over time, perhaps in response to local ecological competitive pressures from other organisms cohabiting the nasopharyngeal space, as well as selection pressures from antibiotics and PCVs (146).

Vaccines and antibiotics are considered the most effective methods against *S. pneumoniae*. Pneumococcal immunization was powered by WHO to prevent *S. pneumoniae* infections. A reduction in CAP in more than 40% after introduction of pneumococcal conjugate vaccine (PCV7) has been reported (147). Recent data on serotypes identified in bacteremia pneumonia in children from Italy since the introduction of PCV7, found serotypes 1 and 19A to be the most common (148). In children under 2 years, all trials have consistently shown a decrease in radiologically-confirmed pneumonia from 23% in the Philippines using PCV11 (149), to 37% in the Gambia with PCV9 (150), and 23.4% in California with PCV7 (151). This effect is most striking in the first year in these three studies. A Cochrane systematic review found a pooled vaccine efficacy for PCV11 of 27% for reduction of radiographically-confirmed pneumonia in children <2 years and 6% for clinical pneumonia (152).

Before the implementation of PCVs, a few pneumococcal serotypes (mainly serotypes 1, 3, 5, 7F, 14, and 19A) were implicated in proven pneumococcal pneumonia and empyema in children (153). The implementation of PCV7 led to a transient reduction in the frequency of CAP (154), rapidly followed by an increase in CAP with pleural effusion and empyema (155), mainly due to serotypes 1 and 7F, and an increase in frequency of serotype 19A, all non-PC7 serotypes. When PCV13, which included these additional serotypes, replaced PCV7, the frequency of both CAP and empyema greatly decreased worldwide (156).

Serotypes 3 and 19A were most closely associated with pneumococcal necrotizing pneumonia. Serotype 3 has a very thick capsule, which strongly resists opsono-phagocytosis and induces a marked inflammatory response, including an intense neutrophilic infiltration with suppurative necrosis (157). In contrast, serotype 19A strains have greater invasive potential, may have a growth advantage over other pneumococcal serotypes in normally sterile sites, and are often resistant to multiple antibiotics (158).

However, with a recent increase in its frequency due to highly invasive non-PCV13 serotypes in Europe, and in pneumococcal meningitis in France, the serotype replacement has raised concerns about the long-term outcome of PCV13 use beyond 5 years after its implementation (156).

## Old Known With New Presentations

### Rhinovirus

Rhinovirus infections are seen worldwide and during all year, with a peak in spring (159). There are 3 species and more than 100 serotypes, so even if the immune response is cross reactive, reinfection is frequent (159). Classically rhinovirus infects the upper respiratory epithelium manifesting as a common cold. Otitis media and rhinosinusitis can also be seen in rhinovirus infection and can be complicated with bacterial coinfection. But, with the development of molecular techniques, it was demonstrated that asymptomatic infection could happen (160). Asymptomatic infection seems to be more frequent in younger children.

#### Clinical Manifestation

Rhinovirus infection can also compromise lower respiratory tract, as bronchiolitis in younger children. Exacerbation of obstructive symptoms can be observed in asthmatic subjects with rhinovirus infection. However, other factors are involved in the development of wheezing in these children, as the inflammatory response, the virus serotypes, among others (161). Otherwise, community acquired pneumonia are also described in rhinoviral infection. A recent Brazilian study demonstrated that in children hospitalized with radiographically diagnosed pneumonia, 43% presented at least one virus and among them rhinovirus was the most frequently detected (162). Importantly, rhinovirus was associated with mild pneumonia, contrarily to respiratory syncytial virus or influenza infection that were associated with more severe cases. It is not uncommon to find concomitantly a second virus with rhinovirus detection, raising the problem of the role that each virus has in the disease (160).

In the last years, with the increased number of immunocompromised patients and the development of neonatology, rhinovirus has acquired a new pathogenic role producing severe pneumonia and even death in some high-risk patients. Patients with cancer or undergoing hematopoietic stem cell transplant receiving corticosteroids or immunosuppressive drugs are at a high risk of developing severe viral pneumonia with prolonged viral shedding (163). These immunosuppressed patients with rhinovirus infection present variable symptoms and severity, but the disease is associated with high morbidity, reaching a mortality rate close to 10% (164). Another group at high risk of severe pneumonia are preterm infants. Two series of preterm infants with rhinovirus infection showed that the majority had respiratory signs such as cough, apnea, or even respiratory distress, requiring respiratory assistance (165, 166).

Special attention is necessary in hospital settings, as nosocomial outbreaks are described and are associated with increased morbidity in high-risk patients (165, 167).

#### Treatment

Management of rhinovirus pneumonia is supportive. The use of pegylated interferon-α2A and ribavirin to control viral replication is described only in case report, without systematic studies (168). Prevention of this infection in high-risk patients is crucial to avoid morbidity and mortality in these particular populations.

### Coronavirus

Four species of coronavirus exist, but human coronavirus belong only to two of them (alphacoronavirus as HCoV-229E and HCoV-NL63 and betacoronavirus as HCoV-OC43, HCoV-HKU1, SARS-CoV-1, MERS-CoV, and SARS-CoV-2) (160). Even though coronavirus circulates globally and throughout the year, some of them are more detected in winter as HCoV-229E, meanwhile others, such as HCoV-NL63, are transmitted more in early summer (169). As influenza viruses, they have human and animal reservoirs. When animal coronavirus is transmitted to humans, an epidemic or pandemic of a new coronavirus can develop with the potential to produce a severe acute respiratory syndrome, as in the novel SARS-CoV-2 (169).

#### Clinical Manifestation

As rhinovirus, “non-SARS” coronaviruses cause common cold and upper respiratory infection. However, lower tract infection has also been related with coronavirus infection. In Thailand, a study detected up to 5.9% of coronavirus infection (229E, OC43, N63, and HKU1) in healthy children hospitalized with community-acquired viral pneumonia (170). Interestingly, in 2.1% of asymptomatic children was also identified coronavirus infection. Similar results were observed in a recent study, identifying coronavirus infection in 9% of children hospitalized with respiratory tract infection (73% of which were lower tract infection) and 10% of asymptomatic controls (171). But higher viral load obtained in symptomatic children supports the role of coronavirus in these respiratory tract infections. Mean hospitalization rate of children younger than 1 year old with low respiratory tract infection and coronavirus detection was 2.8 per 1000 children. Frequently, co-viral detection was identified.

More severe diseases are observed in immunocompromised individuals. A recent study found 2 main risk factors associated with severe low respiratory tract infection: immunocompromised status and viral coinfection (172). Neonatal coronavirus infection has also been associated with increased morbidity, presenting bradycardia and oxygen supplementation (173). These results were observed in a context of nosocomial outbreak, highlighting the importance of prevention and identification of patients at high-risk to develop severe pneumonia.

#### Treatment

Until now, there is no specific antiviral treatment, only supportive care.

## Conclusion

Several emerging pathogens have been described in recent years, with SARS-CoV-2 being responsible for the latest pandemic COVID19. Children are affected just like adults, although the frequency, severity and mortality differ from them in many cases, depending on the causal agent.

Vaccination has been an important ally in reducing the risk of re-emergence pathogens such as measles and pneumococcal infections, however low vaccination coverage and the emergence of serotypes not included in vaccines have favored their re-emergence, with children being the most affected age group.

The antimicrobial resistance, mainly due to unregulated or indiscriminate use of antimicrobials, has led to the emergence of pathogens, mainly in hospital settings, capable of causing pneumonia in children with greater severity due to the delayed onset of effective therapy against these pathogens.

Finally, pathogens that have emerged *de novo* affect children apparently in a lower proportion and less severity than in adults, especially those over 60 years old, who are the most seriously compromised. Avian influenza H5N1 and ANDV in the hantavirus group, are however the exception which cause severe infections with high mortality over 20% in children.

Proper use of antimicrobials and childhood vaccination are and will continue to be highly effective strategies in reducing the risk of pneumonia-causing pathogens in children. Similarly, the rapid international response in identifying emerging infections, the ability to diagnose, sequence and characterize these new pathogens with potential pandemic have been able to contain outbreaks in most of them, even though for COVID19 containment hope is placed on vaccines developed in an incredible short period of time to achieve pandemic control.

Being aware to suspect any of the pathogens discussed here in children, depending on their epidemiological conditions and clinical characteristics, will allow a rapid diagnosis and timely treatment, essential conditions to reduce the risk of complications and mortality from these emerging pathogens.

## Author Contributions

JC-R conceptualized and designed the study. CP and NL collaborated with the study design. CP wrote the first draft. All authors wrote several sections of the paper. All authors read and approved the final manuscript.

## Conflict of Interest

The authors declare that the research was conducted in the absence of any commercial or financial relationships that could be construed as a potential conflict of interest.

## References

[1] Budnik I Ferrés M Pardo T Edwards J Labarca G Reyes F. Contribution of molecular biology in the diagnosis of acute respiratory infections. Rev Chil Enferm Respir. (2016) 32:224–3. 10.4067/S0717-73482016000400003

[2] Chen Z Boon SS Wang MH Chan RWY Chan PKS. Genomic and evolutionary comparison between SARS-CoV-2 and other human coronaviruses. J Virol Methods. (2020) 289:114032. 10.1016/j.jviromet.2020.114032 33290786 PMC7718587

[3] Peiris JS Lai ST Poon LL Guan Y Yam LY Lim W. Coronavirus as a possible cause of severe acute respiratory syndrome. Lancet. (2003) 361:1319–25. 10.1016/S0140-6736(03)13077-2 12711465 PMC7112372

[4] Ksiazek TG Erdman D Goldsmith CS Zaki SR Peret T Emery S. A novel coronavirus associated with severe acute respiratory syndrome. N Engl J Med. (2003) 348:1953–66. 10.1056/NEJMoa030781 12690092

[5] Leung TF Wong GWK Hon KLE Fok TF. Severe acute respiratory syndrome (SARS) in children: epidemiology, presentation and management. Paediatr Respir Rev. (2003) 4:334–9. 10.1016/S1526-0542(03)00088-5 14629957 PMC7129327

[6] Zhong NS Wong GW. Epidemiology of severe acute respiratory syndrome (SARS): adults and children. Paediatr Respir Rev. (2004) 5:270–4. 10.1016/j.prrv.2004.07.011 15531250 PMC7106189

[7] Hon KLE Leung CW Cheng WTF Chan PKS Chu WCW Kwan YW. Clinical presentations and outcome of severe acute respiratory syndrome in children. Lancet. (2003) 361:1701–3. 10.1016/S0140-6736(03)13364-8 12767737 PMC7112484

[8] Leung CW Kwan YW Ko PW Chiu SS Loung PY Fong NC. Severe acute respiratory syndrome among children. Pediatrics. (2004) 113:e535–43. 10.1542/peds.113.6.e535 15173534

[9] Li AM So HK Chu W Ng PC Hon KL Chiu WK. Radiological and pulmonary function outcomes of children with SARS. Pediatric Pulmonol. (2004) 38:427–33. 10.1002/ppul.20078 15514972 PMC7167621

[10] Babyn PS Chu WC Tsou IY Wansaicheong GK Allen U Bitnun A. Severe acute respiratory syndrome (SARS): chest radiographic features in children. Radiol Pediatrician. (2004) 34:47–58. 10.1007/s00247-003-1081-8 14624321 PMC7080132

[11] WHO reports. MERS Situation Update. (2019). Available online at: https://applications.emro.who.int/docs/EMCSR254E.pdf?ua=1 (accessed December 20, 2020).

[12] MacIntyre CR Chen X Adam DC Chughtai AA. Epidemiology of paediatric Middle East respiratory syndrome coronavirus and implications for the control of coronavirus virus disease 2019. J Paediatr Child Health. (2020) 56:1561–4. 10.1111/jpc.15014 32729192 PMC7689819

[13] Zumla A Hui DS Perlman S. Middle East respiratory syndrome. Lancet. (2015) 386:995–1007. 10.1016/S0140-6736(15)60454-8 26049252 PMC4721578

[14] Memish ZA Al-Tawfiq JA Assiri A AlRabiah FA Al Hajjar S Albarrak A. Middle East respiratory syndrome coronavirus disease in children. Pediatric Infect Dis J. (2014) 33:904–6. 10.1097/INF.0000000000000325 24763193

[15] Thabet F Chehab M Bafaqih H Al Mohaimeed S. Middle East respiratory syndrome coronavirus in children. Saudi Med J. (2015) 36:484–6. 10.15537/smj.2015.4.10243 25828287 PMC4404484

[16] Arabi YM Mandourah Y Al-Hameed F Sindi AA Almekhlafi GA Hussein MA. Corticosteroid therapy for critically ill patients with Middle East respiratory syndrome. Am J Respir Crit Care Med. (2018) 197:757–67. 10.1164/rccm.201706-1172OC 29161116

[17] WHO. COVID-19 Weekly Epidemiological Update. Available online at: https://www.who.int/publications/m/item/weekly-epidemiological-update-on-covid-19---8-june-2021

[18] CDC COVID-19 Response Team. Coronavirus Disease 2019 in Children — United States, February 12–April 2, 2020. MMWR Morb Mortal Wkly Rep. (2020) 69:422–6. 10.15585/mmwr.mm6914e4 32271728 PMC7147903

[19] Boban M. Novel coronavirus disease (COVID-19) update on epidemiology, pathogenicity, clinical course and treatments. Int J Clin Pract. (2020) 75:e13868. 10.1111/ijcp.13868 33244856 PMC7744921

[20] Richardson S Hirsch JS Narasimhan M Crawford JM McGinn T Davidson KW. Presenting characteristics, comorbidities, and outcomes among 5700 patients hospitalized with COVID-19 in the New York City area. JAMA. (2020) 323:2052–9. 10.1001/jama.2020.6775 32320003 PMC7177629

[21] Jahangir M Nawaz M Nanjiani D Siddiqui MS. Clinical manifestations and outcomes of COVID-19 in the paediatric population: a systematic review. Hong Kong Med J. (2021) 27:35–45. 10.12809/hkmj208646 32994372

[22] Liguoro I Pilotto C Bonanni M Ferrari ME Pusiol A Nocerino A. SARS-COV-2 infection in children and newborns: a systematic review. Eur J Pediatr. (2020) 179:1029–46. 10.1007/s00431-020-03684-7 32424745 PMC7234446

[23] Tiruneh FT. Clinical profile of Covid-19 in children, review of existing literatures. Pediatric Health Med Ther. (2020) 11:385–92. 10.2147/PHMT.S266063 33061744 PMC7518768

[24] Simoni P Bazzocchi A Boitsios G De Leucio A Preziosi M Aparisi Gómez MP. Chest computed tomography (CT) features in children with reverse transcription-polymerase chain reaction (RT-PCR)-confirmed COVID-19: a systematic review. J Med Imaging Radiat Oncol. (2020) 64:649–59. 10.1111/1754-9485.13098 33000560 PMC7537213

[25] Katal S Johnston SK Johnston JH Gholamrezanezhad A. Imaging findings of SARS-CoV-2 infection in pediatrics: a systematic review of coronavirus disease 2019 (COVID-19) in 850 patients. Acad Radiol. (2020). 27:1608–21. 10.1016/j.acra.2020.07.031 32773328 PMC7392075

[26] Torres JP Izquierdo G Acuña M Pavez D Reyes F Fritis A. Multisystem inflammatory syndrome in children (MIS-C): report of the clinical and epidemiological characteristics of cases in Santiago de Chile during the SARS-CoV-2 pandemic. Int J Infect Dis. (2020) 100:75–81. 10.1016/j.ijid.2020.08.062 32861823 PMC7452906

[27] Fang F Chen Y Zhao D Liu T Huang Y Qiu L. Chinese pediatric society and the editorial committee of the Chinese journal of pediatrics. Recommendations for the diagnosis, prevention, and control of coronavirus disease-19 in children-the Chinese perspectives. Front. Pediatrician. (2020) 8:553394. 10.3389/fped.2020.553394 33224906 PMC7674551

[28] Venturini E Montagnani C Garazzino S Dono D Pierantoni L Lo Vecchio A. Treatment of children with COVID-19: position paper of the Italian Society of Pediatric Infectious Disease. Ital J Pediatrician. (2020) 46:139. 10.1186/s13052-020-00900-w 32972435 PMC7512208

[29] WHO. Influenza at the Human-Animal Interface Summary and Assessment, From 24 October to 9 December. (2020). Available online at: https://www.who.int/influenza/human_animal_interface/Influenza_Summary_IRA_HA_interface_09_12_2020.pdf?ua=1 L(accessed December 27, 2020).

[30] WHO. Cumulative Number of Confirmed Human Cases for A(H5N1) Reported to WHO, 2003-2020. Available online at: https://www.who.int/influenza/human_animal_interface/2020_DEC_tableH5N1.pdf?ua=1. (accessed December 24, 2020).

[31] WHO. Influenza at the Human-Animal Interface Summary and assessment, 13 February to 9 April (2019). Available online at: https://www.who.int/influenza/human_animal_interface/Influenza_Summary_IRA_HA_interface_09_04_2019.pdf?ua=1 (accessed December 24, 2020).

[32] Zhou J Wang D Gao R Zhao B Song J Qi X. Biological features of novel avian influenza A (H7N9) virus. Nature. (2013) 499:500–3. 10.1038/nature12379 23823727

[33] Qin Y Horby PW Tsang TK Chen E Gao L Ou J. Differences in the epidemiology of human cases of avian influenza A(H7N9) and A(H5N1) viruses infection. Clin Infect Dis. (2015) 61:563–71. 10.1093/cid/civ345 25940354 PMC4542598

[34] Sha J Dong W Liu S Chen X Zhao N Luo M. Differences in the epidemiology of childhood infections with avian influenza A H7N9 and H5N1 viruses. PLoS ONE. (2016) 11:e0161925. 10.1371/journal.pone.0161925 27695069 PMC5047462

[35] Oner AF Dogan N Gasimov V Adisasmito W Coker R Chan PK. H5N1 avian influenza in children. Clin Infect Dis. (2012) 55:26–32. 10.1093/cid/cis295 22423125

[36] Bay A Etlik O Oner AF Unal O Arslan H Bora A. Radiological and clinical course of pneumonia in patients with avian influenza H5N1. Eur J Radiol. (2007) 61:245–50. 10.1016/j.ejrad.2006.10.006 17110072

[37] Gao HN Lu HZ Cao B Du B Shang H Gan JH. Clinical findings in 111 cases of influenza A (H7N9) virus infection. N Engl J Med. (2013) 368:2277–85. 10.1056/NEJMoa1305584 23697469

[38] Dai J Zhou X Dong D Liu Y Gu Q Zhu B. Human infection with a novel avian-origin influenza A (H7N9) virus: serial chest radiographic and CT findings. Chin Med J. (2014) 127:2206–11. 24931229

[39] Sivanandy P Zi Xien F Woon Kit L Tze Wei Y Hui En K Chia Lynn L. A review on current trends in the treatment of human infection with H7N9-avian influenza A. J Infect Public Health. (2019) 12:153–8. 10.1016/j.jiph.2018.08.005 30213468

[40] Marjuki H Mishin VP Chesnokov AP De La Cruz JA Davis CT Villanueva JM. Neuraminidase mutations conferring resistance to oseltamivir in influenza A(H7N9) viruses. J Virol. (2015) 89:5419–26. 10.1128/JVI.03513-14 25740997 PMC4442539

[41] Belardo G Cenciarelli O La Frazia S Rossignol JF Santoro MG. Synergistic effect of nitazoxanide with neuraminidase inhibitors against influenza A viruses in vitro. Antimicrob Agents Chemother. (2015) 59:1061–9. 10.1128/AAC.03947-14 25451059 PMC4335909

[42] Haffizulla J Hartman A Hoppers M Resnick H Samudrala S Ginocchio C. A randomized, double-blind, placebo controlled clinical trial of nitazoxanide in adults and adolescents with acute uncomplicated influenza. Lancet Infect Dis. (2014)14:609–18. 10.1016/S1473-3099(14)70717-0 24852376 PMC7164783

[43] Cao B Gao H Zhou B Deng X Hu C Deng C. Adjuvant corticosteroid treatment in adults with influenza A (H7N9) viral pneumonia. Crit Care Med. (2016) 44:e318–28. 10.1097/CCM.0000000000001616 26934144

[44] Ferrés M Vial P Marco C Yañez L Godoy P Castillo C. Prospective evaluation of household contacts of persons with hantavirus cardiopulmonary syndrome in Chile. J Infect Dis. (2007) 195:1563–71. 10.1086/516786 17471425

[45] Padula P Edelstein A Miguel S López N Rossi C Rabinovich R. Hantavirus pulmonary syndrome outbreak in Argentina: molecular evidence for person-to-person transmission of Andes virus. Virology. (1998) 241:323–30. 10.1006/viro.1997.8976 9499807

[46] Martinez-Valdebenito C Calvo M Vial C Mansilla R Marco C Palma RE. Person-to-person household and nosocomial transmission of Andes Hantavirus, Southern Chile, 2011. Emerg Infect Dis. (2014) 20:1637–44. 10.3201/eid2010.140353 25272189 PMC4193174

[47] Ferrés M Martínez-Valdebenito C Angulo J Henríquez C Vera-Otárola J Vergara MJ. Mother-to-child transmission of andes virus through breast milk, Chile. Emerg Infect Dis. (2020) 26:1885–8. 10.3201/eid2608.200204 32687024 PMC7392419

[48] Manigold T Vial P. Human hantavirus infections: epidemiology, clinical features, pathogenesis and immunology. Swiss Med Wkly. (2014) 144:w13937. 10.4414/smw.2014.13937 24652684

[49] Reyes F Ferrés M. Hantavirus: descripción de dos décadas de endemia y su letalidad. ARS MEDICA Rev Cienc Méd. (2018) 43:30–8. 10.11565/arsmed.v44i1.1522

[50] Departamento de Epidemiología Ministerio de Salud de Chile. Boletín Epidemiológico Trimestral HANTAVIRUS. SE 1 – 52, AÑO. (2019). Available online at: http://epi.minsal.cl/wp-content/uploads/2020/02/BET_HANTAVIRUS_2019.pdf (accessed December 29, 2020). 10.33610/23576189.2021.53

[51] Terças-Trettel ACP Melo AVG Bonilha SMF Moraes JM Oliveira RC Guterres A. Hantavirus pulmonary syndrome in children: case report and case series from an endemic area of Brazil. Rev Inst Med Trop Sáo Paulo. (2019) 61:e65. 10.1590/s1678-9946201961065 31859842 PMC6907412

[52] Kumara A Krishnamurthya K Nielsen A. Hantavirus infection among children hospitalized for febrile illness suspected to be dengue in Barbados. J Infect Public Health. (2016) 9:81–7. 10.1016/j.jiph.2015.06.004 26153080

[53] Vial PA Valdivieso F Calvo M Rioseco ML Riquelme R Araneda A. A non-randomized multicentre trial of human immune plasma for treatment of hantavirus cardiopulmonary syndrome caused by Andes virus. Antivir Ther. (2015) 20:377–86. 10.3851/IMP2875 25316807

[54] Guerra FM Bolotin S Lim G Heffernan J Deeks SL Li Y. The basic reproduction number (R_0_) of measles: a systematic review. Lancet Infect Dis. (2017) 17:e420–8. 10.1016/S1473-3099(17)30307-9 28757186

[55] Rota PA Moss WJ Takeda M de Swart RL Thompson KM Goodson JL. Measles. Nat Rev Dis Primers. (2016) 14:16049. 10.1038/nrdp.2016.49 27411684

[56] World Health Organization. New Measles Surveillance Data From WHO. Geneva (2019). Available online at: https://www.who.int/immunization/newsroom/new-measles-data-august-2019/en/

[57] O'Connor P Jankovic D Muscat M Ben-Mamou M Reef S Papania M. Measles and rubella elimination in the WHO Region for Europe: progress and challenges. Clin Microbiol Infect. (2017) 23:504–10. 10.1016/j.cmi.2017.01.003 28111293 PMC6434680

[58] Phadke VK Bednarczyk RA Omer SB. Vaccine refusal and measles outbreaks in the US. JAMA. (2020) 324:1344–5. 10.1001/jama.2020.14828 32797149

[59] Lemos DR Franco AR de Sá Roriz ML Carneiro AK de Oliveira Garcia MH de Souza FL. Measles epidemic in Brazil in the post-elimination period: coordinated response and containment strategies. Vaccine. (2017) 35:1721–8. 10.1016/j.vaccine.2017.02.023 28256359

[60] Patel MK Dumolard L Nedelec Y Sodha SV Steulet C Gacic-Dobo M. Progress toward regional measles elimination — Worldwide, 2000–2018. MMWR Morb Mortal Wkly. Rep. (2019) 68:1105–11. 10.15585/mmwr.mm6848a1 31805033 PMC6897527

[61] Misin A Antonello RM Di Bella S Campisciano G Zanotta N Giacobbe DR. An overview of a re-emerging disease in children and immunocompromised patients. Microorganisms. (2020) 8:276. 10.3390/microorganisms8020276 32085446 PMC7074809

[62] Hester G Nickel A LeBlanc J Carlson R Spaulding AB Kalaskar A. Measles hospitalizations at a United States Children's Hospital 2011-2017. Pediatr Infect Dis J. (2019) 38:547–52. 10.1097/INF.0000000000002221 31117114

[63] Haile T Thach HN Tuan TA Nam DH Dien TM Sato Y. Adenovirus type 7 pneumonia in children who died from measles-associated pneumonia, Hanoi, Vietnam, 2014. Emerg Infect Dis. (2016) 22:687–90. 10.3201/eid2204.151595 26926035 PMC4806935

[64] Lombardo D Ciampi G Spicuzza L. Severe and fatal measles-associated pneumonia during an outbreak in Italy: data from the heart of the epidemic. Adv Respir Med. (2020) 88:197–203. 10.5603/ARM.2020.0118 32706103

[65] Kabra SK Lodha R. Antibiotics for preventing complications in children with measles. Cochrane Database Syst Rev. (2013) 2013:CD001477. 10.1002/14651858.CD001477.pub4 23943263 PMC7055587

[66] World Health Organization [Internet]. Measles Media Factsheet. Available online at: http://www.who.int/mediacentre/factsheets/fs286/en/

[67] Huiming Y Chaomin W Meng M. Vitamin A for treating measles in children. Cochrane Database Syst Rev. (2005) 2005:CD001479. 10.1002/14651858.CD001479.pub2 PMC7076287 16235283

[68] Awotiwon AA Oduwole O Sinha A Okwundu CI. Zinc supplementation for the treatment of measles in children. Cochrane Database Syst Rev. (2017) 6:CD011177. 10.1002/14651858.CD011177.pub3 28631310 PMC6481361

[69] Pai M Behr MA Dowdy D Dheda K Divangahi M Boehme CC. Tuberculosis. Nat Rev Dis Primers. (2016) 2:16076. 10.1038/nrdp.2016.76 27784885

[70] Global Tuberculosis Report 2020. Geneva: World Health Organization. Licence: CC BY-NC-SA 3.0 IGO (2020).

[71] GBD Tuberculosis Collaborators. The global burden of tuberculosis: results from the Global Burden of Disease Study 2015. Lancet Infect Dis. (2018). 18:261–84. 10.1016/S1473-3099(17)30703-X 29223583 PMC5831985

[72] Ford N Shubber Z Meintjes G Grinsztejn B Eholie S Mills EJ. Causes of hospital admission among people living with HIV worldwide: a systematic review and meta-analysis. Lancet HIV. (2015) 2:e438–44. 10.1016/S2352-3018(15)00137-X 26423651

[73] De Lorenzo S Tiberi S. Tuberculosis a re-emerging disease. Intern Emerg Med. (2012) 7(Suppl. 3):S185–7. 10.1007/s11739-012-0822-9 23073855

[74] Seddon JA Johnson S Palmer M van der Zalm MM Lopez-Varela E Hughes J. Multidrug-resistant tuberculosis in children and adolescents: current strategies for prevention and treatment. Expert Rev Respir Med. (2021) 15:221–37. 10.1080/17476348.2021.1828069 32965141

[75] Noguera-Julian A Calzada-HernÁndez J Brinkmann F Basu Roy R Bilogortseva O Buettcher M. Tuberculosis disease in children and adolescents on therapy with anti-tumor necrosis factor-alpha agents: a collaborative, multi-centre ptbnet study. Clin Infect Dis. (2020) 71:2561–9. 10.1093/cid/ciz1138 31796965

[76] Perez-Velez CM Marais BJ. Tuberculosis in children. N Engl J Med. (2012) 367:348–61. 10.1056/NEJMra1008049 22830465

[77] Thomas TA. Tuberculosis in Children. Pediatr Clin North Am. (2017) 64:893–909. 10.1016/j.pcl.2017.03.010 28734517 PMC5555046

[78] Pillay T Andronikou S Zar HJ. Chest imaging in paediatric pulmonary TB. Paediatr Respir Rev. (2020) 36:65–72. 10.1016/j.prrv.2020.10.002 33160839

[79] Kunkel A Abel Zur Wiesch P Nathavitharana RR Marx FM Jenkins HE Cohen T. Smear positivity in paediatric and adult tuberculosis: systematic review and meta-analysis. BMC Infect Dis. (2016) 16:282. 10.1186/s12879-016-1617-9 27296716 PMC4906576

[80] Detjen AK DiNardo AR Leyden J Steingart KR Menzies D Schiller I. Xpert MTB/RIF assay for the diagnosis of pulmonary tuberculosis in children: a systematic review and meta-analysis. Lancet Respir Med. (2015) 3:451–61. 10.1016/S2213-2600(15)00095-8 25812968 PMC4756280

[81] Kay AW Islam SM Wendorf K Westenhouse J Barry PM. Interferon-γ release assay performance for tuberculosis in childhood. Pediatrics. (2018) 141:e20173918. 10.1542/peds.2017-3918 29728429

[82] Guidelines for Treatment of Drug-Susceptible Tuberculosis and Patient Care 2017 Update. Geneva: World Health Organization. Licence: CC BY-NC-SA 3.0 IGO (2017).

[83] Dayan GH Mohamed N Scully IL Cooper D Begier E Eiden J. *Staphylococcus aureus*: the current state of disease, pathophysiology and strategies for prevention. Expert Rev Vaccines. (2016). 11:1373–92. 10.1080/14760584.2016.1179583 27118628

[84] Gillet Y Issartel B Vanhems P Fournet J-C Lina G Bes M. Association between *Staphylococcus aureus* strains carrying gene for Panton-valentine leucocidin and highly lethal necrotising pneumonia in young immunocompetent patients. Lancet. (2002). 359:753–9. 10.1016/S0140-6736(02)07877-7 11888586

[85] Chen J Luo Y Zhang S Liang Z Wang Y Zhang T. Community acquired necrotizing pneumonia caused by methicillin-resistant *Staphylococcus aureus* producing Panton-valentine leucocidin in a Chinese teenager: case report and literature review. Int J Infect Dis. (2014) 26:17–21. 10.1016/j.ijid.2014.02.025 24980464

[86] Schwartz KL Nourse C. Panton-valentine leucocidin-associated *Staphylococcus aureus* necrotizing pneumonia in infants: a report of four cases and review of the literature. Eur J Pediatr. (2012) 171:711–7. 10.1007/s00431-011-1651-y 22159957

[87] Löffler B Niemann S Ehrhardt C Horn D Lanckohr C Lina G. Pathogenesis of *Staphylococcus aureus* necrotizing pneumonia: the role of PVL and an influenza coinfection. Expert Rev Anti Infect Ther. (2013) 11:1041–51. 10.1586/14787210.2013.827891 24073746

[88] Gillet Y Vanhems P Lina G Bes M Vandenesch F Floret D. Factors predicting mortality in necrotizing community-acquired pneumonia caused by *Staphylococcus aureus* containing Panton-valentine leucocidin. Clin Infect Dis. (2007). 45:315–21. 10.1086/519263 17599308

[89] Loffler B Hussain M Grundmeler M Brück M Holzinger D Varga G. *Staphylococcus aureus* Panton-Valentine leukocidin is a very potent cytotoxic factor for human neutrophils. PLoS Pathog. (2010) 6:e1000715. 10.1371/journal.ppat.1000715 20072612 PMC2798753

[90] Shallcross LJ Fragaszy E Johnson AM Hayward AC. The role of the Panton-valentine leucocidin toxin in staphylococcal disease: a systematic review and meta-analysis. Lancet Infect Dis. (2013). 13:43–54. 10.1016/S1473-3099(12)70238-4 23103172 PMC3530297

[91] Geng W Yang Y Wu D Zhang W Wang C Shang Y. Community acquired, methicillin-resistant *Staphylococcus aureus* isolated from children with community-onset pneumonia in China. Pediatr Pulmonol. (2010). 45:387–94. 10.1002/ppul.21202 20232474

[92] Chua K Laurent F Coombs G Grayson ML Howden BP. Not community associated methicillin-resistant *Staphylococcus aureus* (CA-MRSA) A clinician's guide to community MRSA – its evolving antimicrobial resistance and implications for therapy. Clin Infect Dis. (2011). 52:99–114. 10.1093/cid/ciq067 21148528

[93] Sicot N Khanafer N Meyssonnier V Dumitrescu O Tristan A Bes M. Methicillin resistance is not a predictor of severity in community-acquired *Staphylococcus aureus* necrotizing pneumonia – results of a prospective observational study. Clin Microbiol Infect. (2013). 19:E142–8. 10.1111/1469-0691.12022 23237492

[94] Tong SYC Davis JS Eichenberger E Holland TL Fowler VG. *Staphylococcus aureus* infections: epidemiology, pathophysiology, clinical manifestations, and management. Clin Microbiol Rev. (2015). 28:603–61. 10.1128/CMR.00134-14 26016486 PMC4451395

[95] Yang HL Tambyah PA. Fatal bacteraemic pneumonia due to community-acquired methicillin-resistant *Staphylococcus aureus*. Singapore Med J. (2006) 47:1010–1. 17075677

[96] Nimmo GR Coombs GW. Community-associated methicillin resistant *Staphylococcus aureus* (MRSA) in Australia. Int J Antimicrob Agents. (2008) 31:401–10. 10.1016/j.ijantimicag.2007.08.011 18342492

[97] Udo EE Pearman JW Grubb WB. Genetic analysis of community isolates of methicillin-resistant *Staphylococcus aureus* in Western Australia. J Hosp Infect. (1993) 25:97–108. 10.1016/0195-6701(93)90100-E 7903093

[98] Coombs GW Nimmo GR Pearson JC Christiansen KJ Bell JM Collignon PJ. Prevalence of MRSA strains among *Staphylococcus aureus* isolated from outpatients, 2006. Commun Dis Intell Q Rep. (2009) 33:10–20. 19618763 10.33321/cdi.2009.33.2

[99] Rubinstein E Kollef MH Nathwani D. Pneumonia caused by methicillin-resistant *Staphylococcus aureus*. Clin Infect Dis. (2008) 46:S378–85. 10.1086/533594 18462093

[100] Francis JS Doherty MC Lopatin U Johnston CP Sinha G Ross T. Severe community on set pneumonia in healthy adults caused by methicillin resistant *Staphylococcus aureus* carrying the panton-valentine leukocidin genes. Clin Infect Dis. (2005) 40:100–7. 10.1086/427148 15614698

[101] Foster TJ Höök M. Surface protein adhesins of *Staphylococcus aureus*. Trends Microbiol. (1998) 6:484–8. 10.1016/S0966-842X(98)01400-0 10036727

[102] Foster TJ. Immune evasion by staphylococci. Nat Rev Microbiol. (2005) 3:948–58. 10.1038/nrmicro1289 16322743

[103] Hoppe PA Holzhauer S Lala B Bührer C Gratopp A Hanitsch LG. Severe infections of Panton-Valentine leukocidin positive *Staphylococcus aureus* in children. Medicine. (2019) 98:e17185. 10.1097/MD.0000000000017185 31567961 PMC6756729

[104] Khan A Wilson B Gould IM. Current and future treatment options for community-associated MRSA infection. Expert Opin Pharmacother. (2018) 19:457–70. 10.1080/14656566.2018.1442826 29480032

[105] Michelow IC Olsen K Lozano J Rollins NK Duffy LB Ziegler T. Epidemiology and clinical characteristics of community-acquired pneumonia in hospitalized children. Pediatrics. (2004) 113:701–7. 10.1542/peds.113.4.701 15060215

[106] Sztrymf B Jacobs F Fichet J Hamzaoui O Prat D Avenel A. *Mycoplasma*-related pneumonia: a rare cause of acute respiratory distress syndrome (ARDS) and of potential antibiotic resistance. Rev Mal Respir. (2013) 30:77–80. 10.1016/j.rmr.2012.06.012 23318194

[107] Koichi I. Clinical features of severe or fatal *Mycoplasma pneumoniae* pneumonia. Front Microbiol. (2016) 7:800. 10.3389/fmicb.2016.00800 27313568 PMC4888638

[108] Yu JL Song QF Xie ZW Jiang WH Chen JH Fan HF. An iTRAQ-based quantitative proteomics study of refractory *Mycoplasma pneumoniae* pneumonia patients. Jpn J Infect Dis. (2017) 70:571–8. 10.7883/yoken.JJID.2016.355 28003598

[109] Waites KB Balish MF Atkinson TP. New insights into the pathogenesis and detection of *Mycoplasma pneumoniae* infections. Future Microbiol. (2008) 3:635–48. 10.2217/17460913.3.6.635 19072181 PMC2633477

[110] Song Q Xu BP Shen KL. Effects of bacterial and viral co-infections of *Mycoplasma pneumoniae* pneumonia in children: analysis report from Beijing Children's hospital between 2010 and 2014. Int J Clin Exp Med. (2015) 8:15666–74. 26629061 PMC4658950

[111] Zhou Y Wang J Chen W Shen N Tao Y Zhao R. Impact of viral coinfection and macrolide-resistant mycoplasma infection in children with refractory *Mycoplasma pneumoniae* pneumonia. BMC Infect Dis. (2020) 20:633. 10.1186/s12879-020-05356-1 32847534 PMC7447613

[112] Morozumi M Takahashi T Ubukata K. Macrolide-resistant *Mycoplasma pneumoniae:* characteristics of isolates and clinical aspects of community-acquired pneumonia. J Infect Chemother. (2010) 16:78–86. 10.1007/s10156-009-0021-4 20094751

[113] Qu J Chen S Bao F Gu L Cao B. Molecular characterization and analysis of *Mycoplasma pneumoniae* among patients of all ages with community-acquired pneumonia during an epidemic in China. Int J Infect Dis. (2019) 83:26–31. 10.1016/j.ijid.2019.03.028 30926541

[114] Wu PS Chang LY Lin HC Chi H Hsieh YC Huang YC. Epidemiology and clinical manifestations of children with macrolide-resistant *Mycoplasma pneumoniae* pneumonia in Taiwan. Pediatr Pulmonol. (2013) 48:904–11. 10.1002/ppul.22706 23169584

[115] Tanaka T Oishi T Miyata I Wakabayashi S Kono M Ono S. Macrolide-resistant *Mycoplasma pneumoniae* infection, Japan, 2008–2015. Emerg Infect Dis. (2017) 23:1703–6. 10.3201/eid2310.170106 28930026 PMC5621555

[116] Okada T Morozumi M Tajima T Hasegawa M Sakata H Ohnari S. Rapid effectiveness of minocycline or doxycycline against macrolide-resistant *Mycoplasma pneumoniae* infection in a 2011 outbreak among Japanese children. Clin Infect Dis. (2012) 55:1642–9. 10.1093/cid/cis784 22972867

[117] Ishiguro N Koseki N Kaiho M Ariga T Kikuta H Togashi T. Therapeutic efficacy of azithromycin, clarithromycin, minocycline and tosufloxacin against macrolide-resistant and macrolide-sensitive *Mycoplasma pneumoniae* pneumonia in pediatric patients. PLoS ONE. (2017) 12:e0173635. 10.1371/journal.pone.0173635 28288170 PMC5348022

[118] Amaya-Villar R Garnacho-Montero J. How should we treat *Acinetobacter* pneumonia? Curr Opin Crit Care. (2019) 25:465–72. 10.1097/MCC.0000000000000649 31335380

[119] Peleg AY de Breij A Adams MD Cerqueira GM Mocali S Galardini M. The success of Acinetobacter species; genetic, metabolic and virulence attributes. PLoS ONE. (2012) 7:e46984. 10.1371/journal.pone.0046984 23144699 PMC3483291

[120] Garnacho-Montero J Amaya-Villar R. Multiresistant *Acinetobacter baumannii* infections: epidemiology and management. Curr Opin Infect Dis. (2010) 23:332–9. 10.1097/QCO.0b013e32833ae38b 20581674

[121] El Solh AA Alhajhusain A. Update on the treatment of *Pseudomonas aeruginosa* pneumonia. J Antimicrob Chemother. (2009) 64:229–38. 10.1093/jac/dkp201 19520717

[122] Restrepo MI Babu BL Reyes LF Chalmers JD Soni NJ Sibila O. Burden and risk factors for *Pseudomonas aeruginosa* community-acquired pneumonia: a multinational point prevalence study of hospitalized patients. Eur Respir J. (2018) 52:170–90. 10.1183/13993003.01190-2017 29976651

[123] Micek ST Wunderink RG Kollef MH Chen C Rello J Chastre J. An international multicenter retrospective study of *Pseudomonas aeruginosa* nosocomial pneumonia: impact of multidrug resistance. Crit Care. (2015) 19:219. 10.1186/s13054-015-0926-5 25944081 PMC4446947

[124] Jones RN. Microbial etiologies of hospital-acquired bacterial pneumonia and ventilator-associated bacterial pneumonia. Clin Infect Dis. (2020) 51(Suppl. 1):S81–7. 10.1086/653053 20597676

[125] Folgori L Livadiotti S Carletti M Bielicki J Pontrelli G Ciofi Degli Atti ML. Epidemiology and clinical outcomes of multidrug-resistant, gram-negative bloodstream infections in a European tertiary pediatric hospital during a 12-month period. Pediatr Infect Dis J. (2014) 33:929–32. 10.1097/INF.0000000000000339 24642515

[126] Zhang Q Smith JC Zhu Q Guo Z MacDonald NE. A five-year review of *Pseudomonas aeruginosa* bacteremia in children hospitalized at a single center in southern China. Int J Infect Dis. (2012) 16:e628–32. 10.1016/j.ijid.2012.03.014 22709682

[127] Ciofi Degli Atti M Bernaschi P Carletti M Luzzi I Garcia-Fernandez A Bertaina A. An outbreak of extremely drug-resistant *Pseudomonas aeruginosa* in a tertiary care pediatric hospital in Italy. BMC Infect Dis. (2014) 14:494. 10.1186/1471-2334-14-494 25209325 PMC4167521

[128] Langton Hewer SC Smyth AR. Antibiotic strategies for eradicating *Pseudomonas aeruginosa* in people with cystic fibrosis. Cochrane Database Syst Rev. (2014) 11:CD004197. 10.1002/14651858.CD004197.pub4 25383937

[129] Adegoke AA Stenstrom TA Okoh AI. *Stenotrophomonas maltophilia* as an emerging ubiquitous pathogen: looking beyond contemporary antibiotic therapy. Front Microbiol. (2017) 8:2276. 10.3389/fmicb.2017.02276 29250041 PMC5714879

[130] Looney WJ Narita M Muhlemann K. *Stenotrophomonas maltophilia*: an emerging opportunist human pathogen. Lancet Infect Dis. (2009) 9:312–23. 10.1016/S1473-3099(09)70083-0 19393961

[131] Furuichi M Ito K Miyairi I. Characteristics of *Stenotrophomonas maltophilia* bacteremia in children. Pediatr Int. (2016) 58:113–8. 10.1111/ped.12745 26139084

[132] Garcia-Leon G Ruiz de Alegria Puig C Garcia de la Fuente C Martinez-Martinez L Martinez JL Sanchez MB. High-level quinolone resistance is associated with the overexpression of smeVWX in *Stenotrophomonas maltophilia* clinical isolates. Clin Microbiol Infect. (2015) 21:464–7. 10.1016/j.cmi.2015.01.007 25753190

[133] Jeon YD Jeong WY Kim MH Jung IY Ahn MY Ann HW. Risk factors for mortality in patients with *Stenotrophomonas maltophilia* bacteremia. Medicine. (2016) 95:e4375. 10.1097/MD.0000000000004375 27495046 PMC4979800

[134] Ebara H Hagiya H Haruki Y Kondo E Otsuka F. Clinical characteristics of *Stenotrophomonas maltophilia* bacteremia: a regional report and a review of a Japanese case series. Intern Med. (2017) 56:137–42. 10.2169/internalmedicine.56.6141 28090041 PMC5337456

[135] Juhasz E Krizsan G Lengyel G Grosz G Pongracz J Kristof. Infection and colonization by *Stenotrophomonas maltophilia*: antimicrobial susceptibility and clinical background of strains isolated at a tertiary care centre in Hungary. Ann Clin Microbiol Antimicrob. (2014) 13:333. 10.1186/s12941-014-0058-9 25551459 PMC4307884

[136] Mutlu M Yilmaz G Aslan Y Bayramoglu G. Risk factors and clinical characteristics of *Stenotrophomonas maltophilia* infections in neonates. J Microbiol Immunol Infect. (2011) 44:467–72. 10.1016/j.jmii.2011.04.014 21606009

[137] Lai CH Chi CY Chen HP Chen TL Lai CJ Fung CP. Clinical characteristics and prognostic factors of patients with *Stenotrophomonas maltophilia* bacteremia. J Microbiol Immunol Infect. (2004) 37:350–8. 15599467

[138] Friedman ND Korman TM Fairley CK Franklin JC Spelman DW. Bacteraemia due to *Stenotrophomonas maltophilia*: an analysis of 45 episodes. J Infect. (2002) 45:47–53. 10.1053/jinf.2002.0978 12217732

[139] World Health Organization. Pneumonia-Key Factors. Available online at: https//www.who.int/news-room/fact-sheets/details/pneumonia.

[140] GBD 2016. Lower Respiratory Infections Collaborators. Estimates of the global, regional, and national morbidity, mortality, and aetiologies of lower respiratory infections in 195 countries, 1990-2016: a systematic analysis for the Global Burden of Disease Study 2016. Lancet Infect Dis. (2018) 18:1191–210. 10.1016/S1473-3099(18)30310-4 30243584 PMC6202443

[141] Eastham KM Freeman R Kearns AM Eltringham G Clark J Leeming J. Clinical features, aetiology and outcome of empyema in children in the north east of England. Thorax. (2004) 59:522e5. 10.1136/thx.2003.016105 15170039 PMC1747032

[142] Masters IB Isles AF Grimwood K. Necrotizing pneumonia: an emerging problem in children? Pneumonia. (2017) 9:11. 10.1186/s41479-017-0035-0 28770121 PMC5525269

[143] Shenoy AT Orihuela CJ. Anatomical site-specific contributions of pneumococcal virulence determinants. Pneumonia. (2016) 8:7. 10.1186/s41479-016-0007-9 27635368 PMC5021320

[144] Cillniz C Amaro R Torres A. Pneumococcal vaccination. Curr Opin Infect Dis. (2016) 29:187–96. 10.1097/QCO.0000000000000246 26779776

[145] Jauneikaite E Tocheva AS Jefferies JM Gladstone RA Faust SN Christodoulides M. Current methods for capsular typing of *Streptococcus pneumoniae*. J Microbiol Methods. (2015) 113:41–9. 10.1016/j.mimet.2015.03.006 25819558

[146] Linares J Ardunay C Pallares R Fenoll A. Changes in antimicrobial resistance, serotypes and genotypes in *Streptococcus pneumoniae* over a 30-year period. Clin Microbiol Infect. (2010) 16:402–10. 10.1111/j.1469-0691.2010.03182.x 20132251

[147] Falup-Pecurariu O. Lessons learnt after the introduction of the seven valent- pneumococcal conjugate vaccine toward broader spectrum conjugate vaccines. Biomed J. (2012) 35:450–6. 10.4103/2319-4170.104409 23442357

[148] Resti M Moriondo M Cortimiglia M Indolfi G Canessa C Becciolini L. Community-acquired bacteremic pneumococcal pneumonia in children: diagnosis and serotyping by real-time polymerase chain reaction using blood samples. Clin Infect Dis. (2010) 51:1042e9. 10.1086/656579 20883110

[149] Lucero MG Nohynek H Williams G Tallo V Simões EA Lupisan S. Efficacy of an 11-valent pneumococcal conjugate vaccine against radiologically confirmed pneumonia among children less than 2 years of age in the Philippines: a randomized, double-blind, placebo controlled trial. Pediatr Infect Dis J. (2009) 28:455e62. 10.1097/INF.0b013e31819637af 19483514

[150] Cutts FT Zaman SM Enwere G Jaffar S Levine OS Okoko JB. Efficacy of nine-valent pneumococcal conjugate vaccine against pneumonia and invasive pneumococcal disease in the Gambia: randomised, double-blind, placebo-controlled trial. Lancet. (2005) 365:1139e46. 10.1016/S0140-6736(05)71876-6 15794968

[151] Black SB Shinefield HR Ling S Hansen J Fireman B Spring D. Effectiveness of heptavalent pneumococcal conjugate vaccine in children younger than five years of age for prevention of pneumonia. Pediatr Infect Dis J. (2002) 21:810e15. 10.1097/00006454-200209000-00005 12352800

[152] Lucero MG Dulalia VE Nillos LT Williams G Parreño RA Nohynek H. Pneumococcal conjugate vaccines for preventing vaccine-type invasive pneumococcal disease and x-ray defined pneumonia in children less than two years of age. Cochrane Database Syst Rev. (2009) 4:CD004977. 10.1002/14651858.CD004977.pub2 19821336 PMC6464899

[153] Angoulvant F Levy C Grimprel E Varon E Lorrot M Biscardi S. Early impact of 13-valent pneumococcal conjugate vaccine on community-acquired pneumonia in children. Clin Infect Dis. (2014) 58:918–24. 10.1093/cid/ciu006 24532543

[154] Byington CL Korgenski K Daly J Ampofo K Pavia A Mason EO. Impact of the pneumococcal conjugate vaccine on pneumococcal parapneumonic empyema. Pediatr Infect Dis J. (2006) 25:250–4. 10.1097/01.inf.0000202137.37642.ab 16511389

[155] Grijalva CG Nuorti JP Zhu Y Griffin MR. Increasing incidence of empyema complicating childhood community-acquired pneumonia in the United States. Clin Infect Dis. (2010) 50:805–13. 10.1086/650573 20166818 PMC4696869

[156] Alicino C Paganino C Orsi A Astengo M Trucchi C Icardi G. The impact of 10-valent and 13-valent pneumococcal conjugate vaccines on hospitalization for pneumonia in children: a systematic review and meta-analysis. Vaccine. (2017) 35:5776–85. 10.1016/j.vaccine.2017.09.005 28911902

[157] Tsai Y-F Ku Y-H. Necrotizing pneumonia: a rare complication of pneumonia requiring special consideration. Curr Opin Pulm Med. (2012) 18:246–52. 10.1097/MCP.0b013e3283521022 22388585

[158] Hsieh Y-C Chi H Chang K-Y Lai S-H Mu J-J Wong K-S. Increase in fitness of *Streptococcus pneumoniae* is associated with the severity of necrotizing pneumonia. Pediatr Infect Dis J. (2015) 34:499–505. 10.1097/INF.0000000000000631 25461475

[159] Jacobs S Lamson D St. George K Walsh T. Human Rhinoviruses. Clin Microbiol Rev. (2013) 26:135–62. 10.1128/CMR.00077-12 23297263 PMC3553670

[160] Greenberg SB. Update on human rhinovirus and coronavirus infections. Semin Respir Crit Care Med. (2016) 37:555–71. 10.1055/s-0036-1584797 27486736 PMC7171723

[161] Jartti T Gern JE. Role of viral infections in the development and exacerbation of asthma in children. J Allergy Clin Immunol. (2017) 140:895–906. 10.1016/j.jaci.2017.08.003 28987219 PMC7172811

[162] Ferreira HLDS Costa KLP Cariolano MS Oliveira GS Felipe KKP Silva ESA. High incidence of rhinovirus infection in children with community-acquired pneumonia from a city in the Brazilian pre-Amazon region. J Med Virol. (2019) 91:1751–8. 10.1002/jmv.25524 31230362 PMC7166869

[163] Hijano DR Maron G Hayden RT. Respiratory viral infections in patients with cancer or undergoing hematopoietic cell transplant. Front Microbiol. (2018) 9:3097. 10.3389/fmicb.2018.03097 30619176 PMC6299032

[164] Fisher BT Danziger-Isakov L Sweet LR Munoz FM Maron G Tuomanen E. A multicenter consortium to define the epidemiology and outcomes of inpatient respiratory viral infections in pediatric hematopoietic stem cell transplant recipients. Pediat Infect Dis J. (2018) 7:275–82. 10.1093/jpids/pix051 29106589 PMC7107490

[165] Steiner M Strassl R Straub J Böhm J Popow-Kraupp T Berger A. Nosocomial rhinovirus infection in preterm infants. Pediatr Infect Dis J. (2012) 31:1302–4. 10.1097/INF.0b013e31826ff939 22926220

[166] van Piggelen RO van Loon AM Krediet TG Verboon-Maciolek MA. Human rhinovirus causes severe infection in preterm infants. Pediat Infect Dis J. (2010) 29:364–5. 10.1097/INF.0b013e3181c6e60f 19935443

[167] Kobayashi H Shinjoh M Sudo K Kato S Morozumi M Koinuma G. Nosocomial infection by human bocavirus and human rhinovirus among paediatric patients with respiratory risks. J Hosp Infect. (2019) 103:341–48. 10.1016/j.jhin.2019.05.002 31078633

[168] Walter JM Wunderink RG. Severe respiratory viral infections: new evidence and changing paradigms. Infect Dis Clin North Am. (2017) 31:455–74. 10.1016/j.idc.2017.05.004 28687214 PMC7347414

[169] Su S Wong G Shi W Liu J Lai ACK Zhou J. Epidemiology, genetic recombination, and pathogenesis of Coronaviruses. Trends Microbiol. (2016) 24:490–502. 10.1016/j.tim.2016.03.003 27012512 PMC7125511

[170] Dare RK Fry AM Chittaganpitch M Sawanpanyalert P Olsen SJ Erdman DD. Human coronavirus infections in rural Thailand: a comprehensive study using real-time reverse-transcription polymerase chain reaction assays. J Infect Dis. (2007) 196:1321–8. 10.1086/521308 17922396 PMC7109921

[171] Heimdal I Moe N Krokstad S Christensen A Skanke LH Nordbø SA. Human Coronavirus in hospitalized children with respiratory tract infections: a 9-Year population-based study from Norway. J Infect Dis. (2019) 219:1198–206. 10.1093/infdis/jiy646 30418633 PMC7107437

[172] Ogimi C Englund JA Bradford MC Qin X Boeckh M Waghmare A. Characteristics and outcomes of coronavirus infection in children: the role of viral factors and an immunocompromised state. J Pediatric Infect Dis Soc. (2019) 8:21–8. 10.1093/jpids/pix093 29447395 PMC6437838

[173] Gagneur A Vallet S Talbot PJ Legrand-Quillien MC Picard B Payan C. Outbreaks of human coronavirus in a pediatric and neonatal intensive care unit. Eur J Pediatr. (2008) 167:1427–34. 10.1007/s00431-008-0687-0 18335238 PMC7087120

